# Reduction of protein disulfide isomerase results in open conformations and stimulates dynamic exchange between structural ensembles

**DOI:** 10.1016/j.jbc.2022.102217

**Published:** 2022-06-30

**Authors:** Mathivanan Chinnaraj, Robert Flaumenhaft, Nicola Pozzi

**Affiliations:** 1 Edward A. Doisy Department of Biochemistry and Molecular Biology, Saint Louis University School of Medicine, St Louis, Missouri, USA; 2 Division of Hemostasis and Thrombosis, Department of Medicine, Beth Israel Deaconess Medical Center, Harvard Medical School, Boston, Massachusetts, USA

**Keywords:** single-molecule FRET, protein dynamics, thiol isomerases, allostery, protein disulfide isomerase, thrombosis, fFCS, filtered fluorescence correlation spectroscopy, HS-AFM, high speed atomic force microscopy, PDA, photon distribution analysis, PDI, protein disulfide isomerase, SAXS, small-angle X-ray scattering, smFRET, single-molecule FRET, TCSPC, time-correlated single photon counting, TNB^2−^, 2-nitro-5-thiobenzoate anion

## Abstract

Human protein disulfide isomerase (PDI) is an essential redox-regulated enzyme required for oxidative protein folding. It comprises four thioredoxin domains, two catalytically active (**a**, **a’**) and two inactive (**b**, **b’**), organized to form a flexible **abb’a’** U-shape. Snapshots of unbound oxidized and reduced PDI have been obtained by X-ray crystallography. Yet, how PDI’s structure changes in response to the redox environment and inhibitor binding remains controversial. Here, we used multiparameter confocal single-molecule FRET to track the movements of the two catalytic domains with high temporal resolution. We found that at equilibrium, PDI visits three structurally distinct conformational ensembles, two “open” (O_1_ and O_2_) and one “closed” (C). We show that the redox environment dictates the time spent in each ensemble and the rate at which they exchange. While oxidized PDI samples O_1_, O_2_, and C more evenly and in a slower fashion, reduced PDI predominantly populates O_1_ and O_2_ and exchanges between them more rapidly, on the submillisecond timescale. These findings were not expected based on crystallographic data. Using mutational analyses, we further demonstrate that the R300-W396 cation-π interaction and active site cysteines dictate, in unexpected ways, how the catalytic domains relocate. Finally, we show that irreversible inhibitors targeting the active sites of reduced PDI did not abolish these protein dynamics but rather shifted the equilibrium toward the closed ensemble. This work introduces a new structural framework that challenges current views of PDI dynamics, helps rationalize its multifaceted role in biology, and should be considered when designing PDI-targeted therapeutics.

Protein disulfide isomerase (PDI) is an archetypal oxidoreductase responsible for oxidative protein folding in eukaryotes. It was the first thiol isomerase to be discovered and is arguably the most important member of this large family (1, 2, 3, 4, 5). It interacts with many client proteins catalyzing the formation, cleavage, and isomerization of disulfide bonds (6). Since disulfide bonds are essential for achieving tertiary and quaternary structures of proteins and have important functional roles (7), its enzymatic activity is essential for life.

The human enzyme, named hereafter PDI, comprises 508 amino acids. From the N terminus, the first 17 amino acids compose the signal peptide. The remaining 491 amino acids are organized in four thioredoxin domains arranged in the order **a**, **b**, **b’**, and **a’**, followed by an acidic C-terminal tail (Fig. 1*A*) (3, 5). Domains **a** and **a’** are catalytically active as they contain the conserved motif CxxC, whereas the **b** and **b’** domains are catalytically inactive. Three interdomain linkers connect the four thioredoxin domains. Of those, the one connecting **b’** and **a’**, also known as the **x**-linker, is the longest and the most flexible (8).

**Figure 1:**
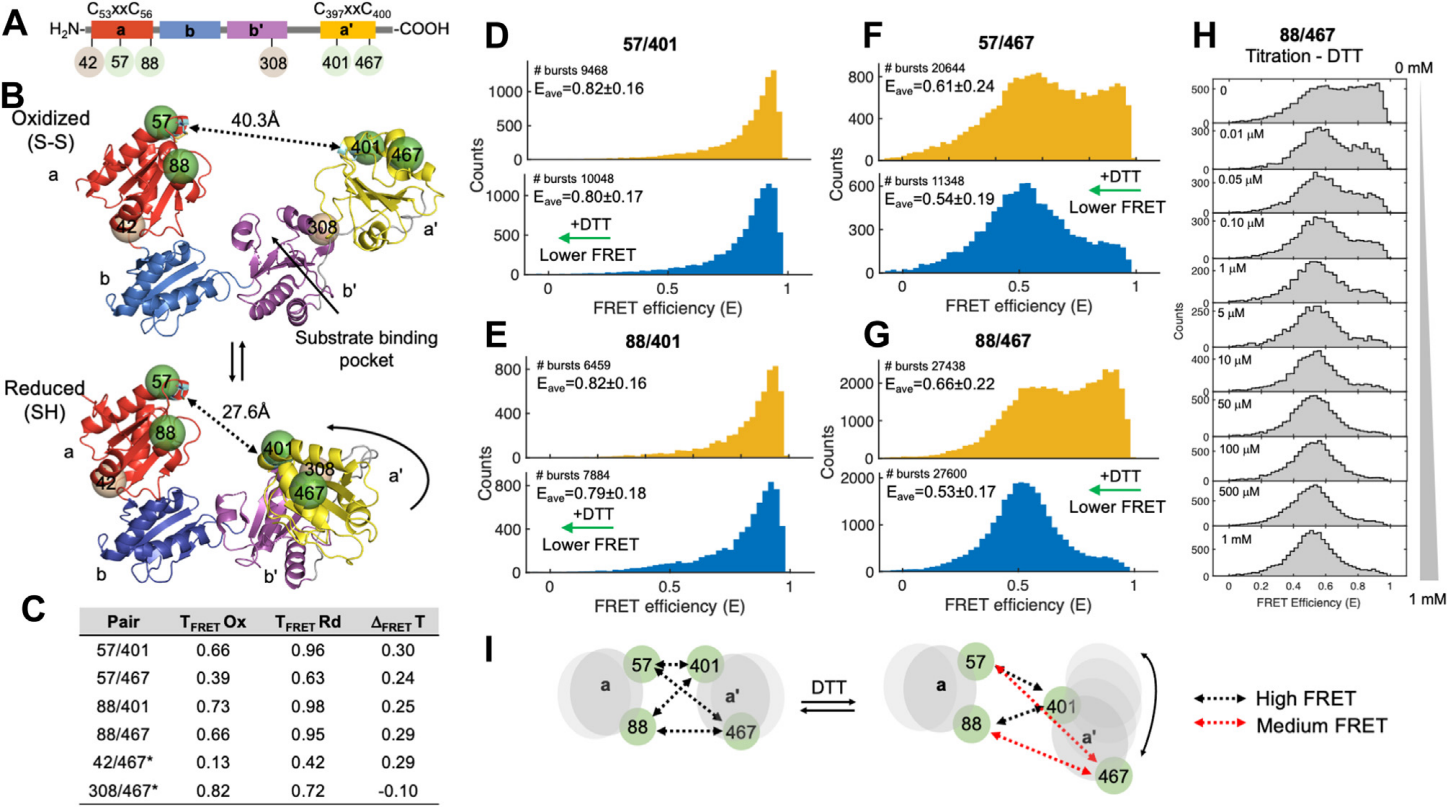
**smFRET studies of oxidized and reduced PDI.***A*, domain structure of PDI showing the location of the active sites (CxxC), new (*green*) and previously published (*gray*) residues selected to incorporate the FRET pairs. *B*, top view of the X-ray crystal structures of oxidized (S-S, top panel, 4el1) and reduced (SH, bottom panel, 4ekz) PDI documenting a U-shape architecture and movement of the a’ domain (*yellow*) toward the b’ domain (*magenta*) upon reduction of the active sites. Shown as *dotted lines* are the distances between the active sites. The *green spheres* represent residues 57, 88, 401, and 467 selected for smFRET studies. *C*, theoretical values of energy transfer for oxidized (T_FRET_Ox) and reduced PDI (T_FRET_Rd) estimated with the software FPS. The difference T_FRET_Ox-T_FRET_Rd is shown as Δ_FRET_. *D* and *E*, FRET histograms of doubly labeled (0.25<S > 0.75) oxidized (*yellow*) and reduced (*blue*) 57/401 (*D*), 88/401 (*E*), 57/467 (*F*), and 88/467 (*G*). Shown are the number of bursts and mean FRET (E_ave_) ± STDEV. Note how, in the presence of DTT, the signal shifts toward lower FRET (*green arrow*). *H*, FRET histograms of 88/467 collected at increasing concentrations of DTT (0–1 mM). To ensure equilibrium, samples were measured after incubating 88/467 and DTT for 40 min at room temperature (20 °C). Experimental conditions are Tris 20 mM pH 7.4, 150 mM NaCl, 2 mM EDTA, 0.003% Tween 20. PDI’s concentration was ∼100 pM. Collection time was ∼40 min per sample. *I*, movements of the catalytic domains that satisfy the FRET data. PDI, protein disulfide isomerase; smFRET, single-molecule FRET.

PDI is primarily located in the endoplasmic reticulum (3) but can also be found outside the cells where it plays key regulatory roles in several physiologic and pathophysiologic processes, most notably in thrombus formation (9). Regardless of the location, because of the motif CxxC, its catalytic activity is critically regulated by the redox environment *via* cysteine modifications, for example, oxidation (S-S) or reduction (-SH). Hence, understanding how the structure of PDI responds to changes in the redox milieu is important for human biology, and particularly, for the field of hematology, as this knowledge could define the mechanistic basis of its extracellular function, hence helping to design compounds capable of targeting redox-specific activities of extracellular PDI for safe anticoagulation.

Over the past 2 decades, structural, computational, and biophysical studies have provided solid evidence for PDI’s flexibility by documenting large-scale redox-dependent and redox-independent movements of the two catalytic domains (10). This led to the hypothesis that PDI operates as a dynamic clamp capable of opening and closing in response to different stimuli. Two single-molecule approaches have been recently applied to the human enzyme to test this hypothesis. Okumura *et al*. used high speed atomic force microscopy (HS-AFM) to image single PDI molecules that were tethered onto mica sheets (11). These studies support the hypothesis that oxidized PDI is more open and dynamic than reduced PDI, which adopts a compact/collapsed conformation. In the meantime, our group developed a method to incorporate unnatural amino acids into PDI (12) and performed proof-of-concept single-molecule FRET (smFRET) investigations of PDI in solution. While confirming PDI’s flexibility reported by Okumura *et al*., our studies suggested that reduced PDI may not be as closed and rigid as previously thought, thus warranting further investigations.

This study describes the conformational dynamics of oxidized and reduced PDI in solution using multiparameter confocal smFRET. We chose this approach because it enables the identification and quantification of large-scale dynamics with a high temporal resolution (μs-ms) (13, 14, 15) while minimizing the probability of structural perturbations caused by the immobilization of proteins onto a surface. Using probes located in the catalytic domains, we discovered that PDI rapidly and spontaneously visits three major conformational ensembles at equilibrium. We showed that the redox environment affects the dwell times in each ensemble but does not elicit new conformations. While movements of the catalytic domains were anticipated based on current literature, how the catalytic domains relocate in response to the redox environment was unexpected, as was the structural role of the R300-W396 cation-π interaction and the active site cysteines. Identification and quantification of submillisecond dynamics led us to propose a new structural model that more soundly explains how PDI might operate as a multifunctional enzyme and may assist drug development efforts.

## Results

### Design of FRET pairs and site-specific labeling

The X-ray crystal structures of oxidized and reduced human PDI were previously solved (16) (Fig. 1*B*). They document a U-shape architecture and movement of the **a’** domain toward the **a** domain upon reduction of the active sites. Since the major substrate-binding pocket is located between the **a** and **a’** domains, movements of the catalytic domains are expected to regulate enzyme function by controlling access/release of substrates to/from the pocket and facilitate the interaction of the active sites with the bound substrate. Notably, molecular dymamics studies performed using these structures as a starting point for the simulations (10, 17), while capturing movements that resemble the ones reported by X-ray crystallography, identified several other motions of the catalytic domains, revealing a vast array of possible structural configurations. Thus, it remains unclear which macroscopic states PDI most frequently visits at equilibrium, how fast these states interconvert, and how the redox environment affects structural flexibility.

In our previous work (12), we generated two FRET pairs, 42/467 and 308/467, the first one reporting on the movements of the **a** and **a’** domains and the latter reporting on the movements of the **b’** and **a’** domains (Fig. 1, *A* and *B*). Surprisingly, we found that reduction of oxidized PDI by the reducing agents DTT and GSH resulted in lower values of FRET, which indicates that reduction of the active site cysteines promotes transition toward structures in which the **a’** domain might be more distant from the **a** and **b’** domains. This was not expected based on X-ray crystallography and HS-AFM, which predict compaction of PDI structure upon reduction (16, 18) but is in line with a recent crystal structure of the microsomal triglyceride transfer protein complex (19), which documents an open conformation of reduced PDI. In this study, we designed four additional combinations of labeling positions, 57/401, 88/467, 57/467, and 88/401 (Fig. 1, *A* and *B*), which track in greater detail the distance between the two catalytic domains, hence the overall conformation that PDI adopts in solution. The new FRET pairs were designed such that, based on the crystal structures, we expect a significantly higher value of energy transfer (ΔFRET>0.2) as PDI transitions from the oxidized (lower FRET) to the reduced state (higher FRET) (Fig. 1*C*).

Since the two catalytic domains contain the motif CxxC, donor and acceptor fluorophores were introduced at the desired positions using biorthogonal chemistry following a procedure recently developed and validated in our laboratory (12). Atto550 (donor) and Atto647N (acceptor) were chosen because of their long fluorescence lifetime and excellent photostability, as these features are necessary to carry out multiparameter confocal smFRET studies. The R_0_ for this FRET pair was determined to be 62.3 Å, which agrees with previous reports (20).

Before carrying out smFRET measurements, structural integrity and catalytic activity were rigorously tested. We found that doubly labeled 57/401, 57/467, 88/401, and 88/467 were monomeric (Fig. S1), properly folded (Fig. S2*A*), capable of sensing changes in redox environment (Fig. S2*B*) and catalytically active (Fig. S2*C*) to a degree consistent with what we expected based on the positioning of the dyes. Specifically, residues 88 and 467 are located >15 Å away from the active site cysteines 56 and 400, whereas residues 57 and 401 are located ∼5 Å away from the active site cysteines 56 and 400. Since incorporation of the dyes at positions 57 and 401, but not at positions 88 and 467, is likely to reduce the accessibility of the substrate to the active sites, we expected doubly labeled 88/467 to be identical to unlabeled 88/467 and PDI WT but different from doubly labeled 88/401, 57/467, and 57/401. Findings in Fig. S2*C* document that the reductase activity of doubly labeled 88/467 and unlabeled 88/401, 57/467, and 57/401 is very similar to PDI WT, thus supporting this view. Doubly labeled PDI variants are therefore suitable for smFRET studies.

### Conformational dynamics of oxidized and reduced PDI

smFRET studies were performed using a confocal microscope equipped with pulse interleaved excitation, enabling the identification and analysis of single molecules properly labeled with donor and acceptor (Figs. S3 and S4). After purification, the active site cysteines were oxidized. This was previously documented using cysteine-reactive maleimide dyes (12) and further confirmed here by Ellman’s reaction (Fig. S5) and reoxidation 3experiments monitored by smFRET (Fig. S6). Importantly, reoxidation experiments prove that the FRET changes induced by DTT are reversible, as expected for a catalytically active enzyme shuffling between oxidized and reduced states.

Under oxidizing conditions, 57/401 (Fig. 1*D*) and 88/401 (Fig. 1*E*) were found to adopt a unimodal distribution skewed toward high FRET. The mean FRET (E_ox_) was 0.82 ± 0.16 for both variants. In contrast, oxidized 57/467 (Fig. 1*F*) and 88/467 (Fig. 1*G*) displayed broad FRET distributions, spanning almost the entire FRET range. This resulted in lower FRET values (E_ox_ =0.61 for 57/467 and E_ox_ =0.66 for 88/467) and larger SDs (0.24 for 57/467 and 0.22 for 88/467).

To transform oxidized PDI into reduced PDI, we added the reducing agent DTT. When 1 mM DTT was added to oxidized PDI, the FRET signal shifted toward lower values for all the FRET pairs. A larger shift was seen for 88/467 (ΔE_(ox-rd)_=0.13) (Fig. 1*G*) and 57/467 (ΔE_(ox-rd)_=0.07) (Fig. 1*F*). A smaller shift was measured for 88/401 (ΔE_(ox-rd)_=0.03) (Fig. 1*E*) and 57/401 (ΔE_(ox-rd)_=0.02) (Fig. 1*D*). This indicates that PDI undergoes a significant structural reorganization in the presence of DTT. Specifically, the catalytic domains move away from each other so that residues 57/401 and residues 88/401 remain, most of the time, significantly closer than residues 88/467 and residues 57/467 (Fig. 1*I*).

Fluorescent dyes could respond to thiols as found in DTT or the active sites of reduced PDI in the form of extended dark state lifetimes (21). This effect could explain why the high FRET state is shifted toward lower FRET upon addition of DTT. In our system, however, this quenching hypothesis is unlikely for the following reasons. First, the molecular brightness of both donor and acceptor was not affected by DTT (Fig. S7). Second, the effect of DTT on 88/467 was dose dependent and saturable, as shown in Figure 1*H*. Third, the fluorescent dyes are attached to PDI using flexible linkers. Based on anisotropy measurements (Table S1), they freely rotate in solution; they are not close to the active sites. Nonetheless, to avoid conclusions based on one set of dyes, we labeled 57/401, 88/401, and 88/467 with a different combination of dyes (*i.e.*, sulfoCy3/sulfoCy5). Consistent with our interpretations, the smFRET profiles of oxidized 57/401, 88/401, and 88/467 were similar to those obtained with Atto dyes, as was the effect of DTT (Fig. S8). Furthermore, addition of DTT caused a greater shift toward lower FRET for 57/401 and 88/401, which was anticipated because of the lower value of R_0_ of the sulfoCy dyes (∼56 Å) (20). Thus, data with the sulfoCy dyes support the conclusions made with the Atto dyes and reveal that the high FRET population in 57/401 and 88/401 is dynamic too.

### Oxidized and reduced PDI undergo rapid conformational dynamics

**Figure 2:**
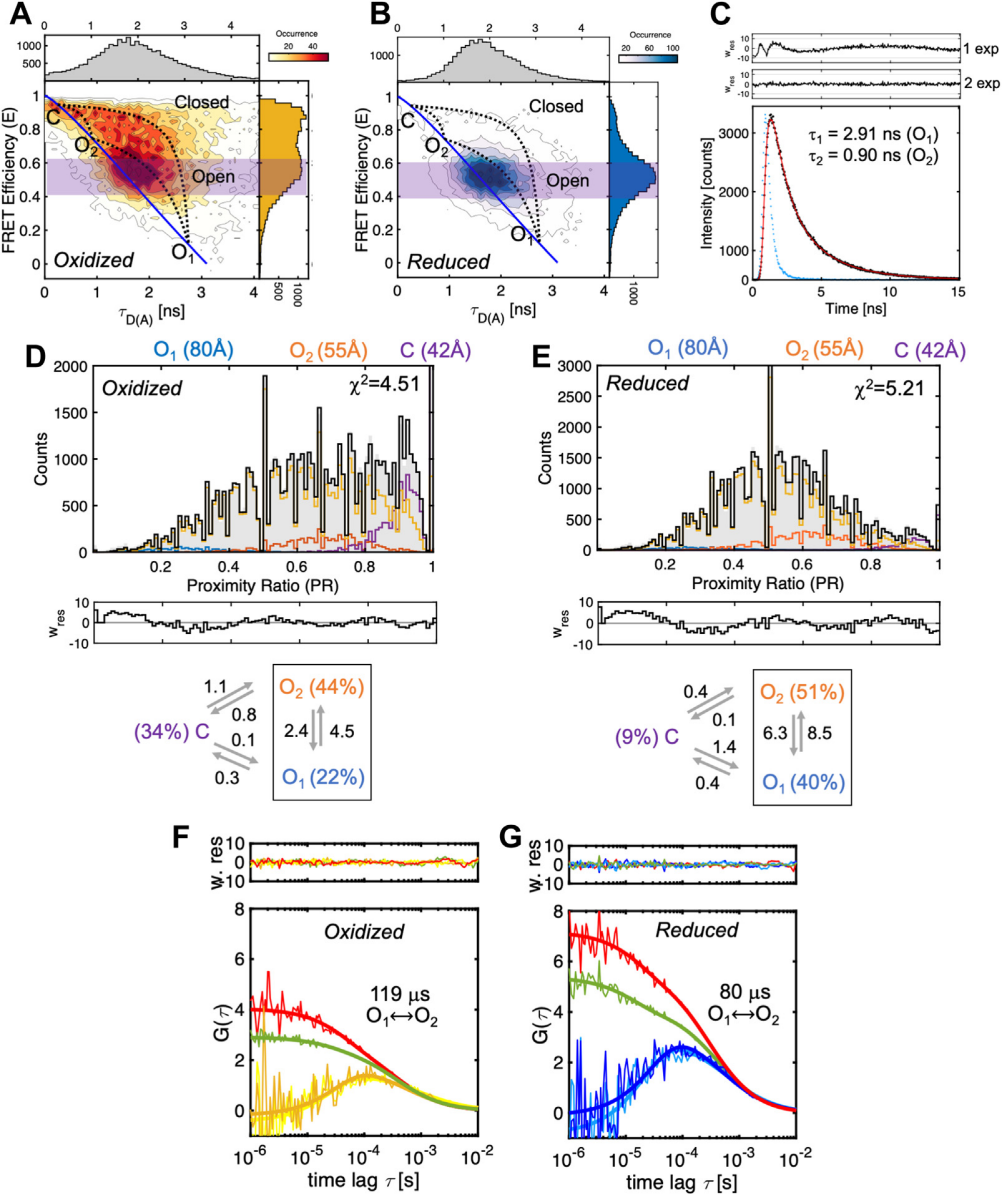
**Fast dynamics of oxidized and reduced PDI.***A* and *B*, plots of FRET efficiency *versus* lifetime of the donor in the presence of the acceptor (τ_D(A)_) for (*A*) oxidized and (*B*) reduced 88/467 documenting dynamic exchange between closed (C, high FRET) and open (O, medium FRET) ensembles. The *solid blue line* describes the static FRET line. Systematic deviations from the static FRET line highlighted by the *dotted lines* track the trajectory of single molecules that are exchanging between O_1_ (state 1), O_2_ (state 2), and C (state 3) while passing through the confocal volume. The *magenta* regions indicate the molecules selected for the lifetime analysis (0.4–0.6) shown in panel (*C*). *C*, subpopulation-specific fluorescence lifetime of oxidized 88/467. Data points are in *black*. The *red line* represents the best fit obtained with a double exponential function (χ^2^=1.33). The value of the lifetime of each population is shown in the plot. It is also reported in Table S2, together with the amplitude for each population. The instrumental response function is shown in *blue*. Weighted residuals for one (1 exp) and two exponential (2 exp) fits are shown above the graph. *D* and *E*, PDA analyses of oxidized (*D*) and reduced (*E*) 88/467 obtained with a dynamic three-state model. C, O_1,_ and O_2_ states are shown in *purple*, *orange*, and *blue*, respectively while the *yellow line* represents the exchange between them. The *black line* represents the global fit. Weighted residuals are shown below each plot, together with a diagram that summarizes the kinetic scheme, the rate constants in ms^−1^, and the fraction of each population at equilibrium. Note how the residuals show a larger deviation toward lower values of PR. Such deviation is likely due to acceptor fluorophore that goes into a dark, non-FRET state. *F* and *G*, rapid exchange between O_1_ and O_2_ ensembles monitored by fFCS. Autocorrelation (*red* and *green*) and crosscorrelation curves (*yellow* and *blue*) of O_1_ and O_2_ ensembles for oxidized (*F*) and reduced (*G*) 88/467. The *solid lines* represent the best fit model obtained by globally fitting the four curves, which enables the extraction of the interconversion time, expressed in microseconds. Randomly distributed weighted residuals are shown above each plot. Best fit parameters are τ_R_,_ox_= 119 ± 12 μs; τ_D_=469 μs (fixed, diffusion), χ^2^=1.11; τ_R_,_rd_ = 80 ± 9 μs; τ_D_=469 μs (fixed, diffusion), χ^2^=1.25. fFCS, filtered fluorescence correlation spectroscopy; PDA, photon distribution analysis; PDI, protein disulfide isomerase.

Broad FRET profiles such as those measured for 57/467 and 88/467 are characteristic of flexible proteins rapidly exchanging between multiple conformations at equilibrium. To explore this possibility, we took advantage of the way photons are collected and stored in our experiments to construct plots in which the transfer efficiency of oxidized and reduced PDI was graphed *versus* the fluorescence lifetime of the donor in the presence of the acceptor (τ_D(A)_) of each molecule. In these plots, as demonstrated elsewhere (13), FRET populations that represent conformational states (or ensembles) that either do not exchange or exchange at a rate ∼10 times slower than the molecules’ diffusion time lie on the so-called “static” FRET line, which is the line that describes the theoretical relationship between the values of τ_D(A)_ and the values of energy transfer. By contrast, FRET populations representing conformational states undergoing dynamic exchange during the observation time deviate from the “static” FRET line and lie on the “dynamic” FRET line, which connects two exchanging states. Plots of oxidized (Fig. 2*A*) and reduced 88/467 (Fig. 2*B*), but also 57/467 (Fig. S9), revealed two main populations of molecules connected by a bridge, thus unequivocally documenting that the broad FRET profiles seen in Figure 1 are due to conformational dynamics unfolding in the millisecond timescale. This is because PDI molecules remain, on average, ∼0.5 ms in the observation volume. For simplicity, we called these two populations closed (C) and open (O), respectively. C and O were indistinguishable in the FRET pairs 57/401 and 88/401 because residues 57, 88, and 401 are closer than ∼45 Å. Nonetheless, 57/401 and 88/401 clearly showed hints of dynamics as bursts deviated from the static FRET line, especially in the presence of DTT (Fig. S9).

After having established that oxidized and reduced PDI are dynamic, we next sought to define the minimum number of macroscopic states at equilibrium. To do so, we focused on 88/467. This is because, in addition to responding well to DTT, labeling at positions 88 and 467 had no effects on secondary structure and reductase activity (Fig. S2*C*). At first, we carefully inspected the plots of 88/467, focusing on where the center of the ensembles lies compared to the static FRET line. In contrast to the closed population, we noticed that the center of the open population did not reside on the static FRET line but was instead shifted toward the right. Since the dyes are freely rotating in solution, thus not theoretically affecting protein dynamics, because of current literature (13, 15, 22), we hypothesized that such deviation from the static FRET line of the open population arises from multiple (at least two) FRET states that exchange very rapidly (submillisecond), faster than diffusion through the open detection volume of ∼1 fl. We performed subpopulation-specific fluorescence lifetime analysis (13, 23) to test this hypothesis. Since we hypothesized heterogeneity within the open ensemble, we selected bursts from the FRET interval 0.4 to 0.6, which is highlighted in *magenta* (Fig. 2, *A* and *B*). If multiple PDI species were present at equilibrium, we expected more than one relaxation would be necessary to fit the lifetime plots. For both oxidized and reduced 88/467 (Fig. 2*C*), the lifetime decay could not be fit with one exponential (1 exp, χ^2^=9.69) but instead required a double exponential function (2 exp, χ^2^=1.31). Adding a third relaxation did not significantly improve the fit (χ^2^=1.28). This result agrees with our hypothesis and documents two states within the open ensemble, which we called O_1_ and O_2_. Interestingly, the ratio f_2_/f_1_ between the amplitudes f_2_ for τ_2_ and f_1_ for τ_1_ was ∼1.2 in both oxidized and reduced PDI. Thus, O_2_ is more represented at equilibrium than O_1_, regardless of the redox state. Also interesting was that the values of τ_1_ and τ_2_ were very similar for oxidized and reduced 88/467, within experimental error (Table S2). This indicates that, despite the obvious differences between the two FRET profiles, three similar, perhaps identical, macroscopic states exist in both redox states. These are C, O_1_, and O_2_.

### Connectivity between the ensembles and rates of interconversion

After having identified the minimum number of macroscopic states at equilibrium, we next defined the connectivity between them. To this end, using the previously determined lifetime values of 0.25 ns for C, 0.91 ns for O_2_, and 2.75 ns for O_1_, we drew the corresponding dynamic FRET lines (*dotted lines*) in Figure 2, *A* and *B*. Although less evident for reduced PDI because of the low intensity of C, we found bursts lying on all three lines indicating dynamic exchange between the FRET states. This led us to propose a triangular kinetic scheme for both reduced and oxidized PDI, implying that, in solution, the three ensembles spontaneously exchange with one another.

To quantify the abundance of each ensemble at equilibrium and determine the rates at which they interconvert, we performed photon distribution analysis (PDA) (22, 24, 25). Given the results of our previous experiments, we chose a three-state dynamic model. Datasets for oxidized and reduced 88/467 binned at 1, 0.75, 0.5, and 0.25 ms (Fig. S10) were globally fit after fixing the value for each state to 42 Å for C, 55 Å for O_2_, and 80 Å for O_1_, which were calculated using the equation E=1-(τ_D(A)_/τ_D_), a τ_D_=3.2 ns and an R_0_ = 62.3 Å, and restricting the value of sigma (σ) to 0.045. Sigma defines the width of a shot-noise limited distribution and was experimentally determined in our system using fluorescently labeled dsDNA constructs with different lengths (Fig. S11). Representative results obtained with datasets binned at 0.5 ms are shown in Figure 2, *D* and *E* for oxidized and reduced 88/467, respectively. The rate constants measured by PDA are reported in Table 1 and summarized in the scheme located below each plot.

**Table 1:** PDA analysis of oxidized (top) and reduced (bottom) PDI 88/467 WT and variants used in this study

Rates (ms^−1^)	WT	AA/AA	CC/AA	AA/CC	R300H	W396A
**k**_**1,2**_	4.50 ± 0.81	2.14 ± 0.14	4.43 ± 0.05	2.73 ± 0.36	1.97 ± 0.41	1.36 ± 0.14
**k**_**1,3**_	0.32 ± 0.31	0.63 ± 0.23	0.34 ± 0.13	0.26 ± 0.28	0.51 ± 0.47	1.38 ± 0.09
**k**_**2,1**_	2.38 ± 0.36	1.59 ± 0.13	2.39 ± 0.09	1.51 ± 0.11	1.35 ± 0.22	1.51 ± 0.39
**k**_**2,3**_	0.78 ± 0.26	1.03 ± 0.09	0.67 ± 0.06	0.68 ± 0.12	0.70 ± 0.55	0.80 ± 0.11
**k**_**3,1**_	0.15 ± 0.11	0.66 ± 0.15	0.12 ± 0.02	0.48 ± 0.24	0.09 ± 0.11	0.13 ± 0.04
**k**_**3,2**_	1.07 ± 0.24	0.95 ± 0.11	0.94 ± 0.06	0.66 ± 0.24	1.20 ± 0.16	0.93 ± 0.16
**% state**						
**O**_**1**_**(80 Å)**	23 ± 1	29 ± 1	22 ± 1	26 ± 1	22 ± 2	20 ± 1
**O**_**2**_**(57 Å)**	44 ± 3	36 ± 2	43 ± 1	42 ± 2	39 ± 4	32 ± 2
**C (42 Å)**	34 ± 1	35 ± 2	35 ± 1	32 ± 1	39 ± 2	49 ± 1
**χ2(global)**	5.5	6.2	5.2	4.9	6.7	4.7


Rates (ms^−1^)	WT	AA/AA	CC/AA	AA/CC	R300H	W396A
**k**_**1,2**_	8.49 ± 1.95	2.12 ± 0.15	9.35 ± 0.30	4.33 ± 1.29	6.64 ± 0.21	4.49 ± 0.62
**k**_**1,3**_	0.36 ± 0.33	0.39 ± 0.34	0.99 ± 0.66	1.03 ± 1.04	0.94 ± 1.29	0.78 ± 0.09
**k**_**2,1**_	6.28 ± 1.07	1.45 ± 0.39	7.50 ± 1.95	3.52 ± 0.32	4.09 ± 0.18	4.72 ± 0.40
**k**_**2,3**_	0.11 ± 0.06	0.75 ± 0.59	0.75 ± 1.19	0.27 ± 0.09	0.17 ± 0.13	0.16 ± 0.05
**k**_**3,1**_	1.39 ± 0.84	0.48 ± 0.17	3.95 ± 4.47	0.79 ± 0.19	0.36 ± 0.55	0.25 ± 0.22
**k**_**3,2**_	0.40 ± 0.21	0.97 ± 0.23	2.05 ± 2.72	0.50 ± 0.50	0.68 ± 0.60	1.30 ± 0.11
**% state**						
**O**_**1**_**(80 Å)**	39 ± 1	28 ± 1	41 ± 1	37 ± 3	33 ± 1	37 ± 1
**O**_**2**_**(57 Å)**	51 ± 3	37 ± 2	49 ± 3	45 ± 6	53 ± 1	40 ± 2
**C (42 Å)**	9 ± 2	35 ± 1	10 ± 2	18 ± 3	14 ± 1	23 ± 1
**χ2(global)**	4.5	5.1	4.2	5.2	6.1	5.1

PDA was performed on datasets binned at 0.25, 0.5, 0.75, and 1 ms. To assess robustness of the fit, the PDA fit was repeated by systematically varying the initial value of the rate constants to 1, 0.5, and 0.75 ms^−1^ (min 0, max 10) while keeping the other settings identical. The results in the tables above represent the average of the three independent determinations.

The most notable difference between the two redox states identified by PDA concerns the distribution of the three ensembles at equilibrium. In the presence of DTT, C was minimally populated (∼9%), whereas O_1_ and O_2_ accounted for ∼40% and ∼51%, respectively. By contrast, oxidized PDI spent a similar amount of time in C and O_1_ but preferred O_2_. The fact that O_2_ dominates agrees with our previous analysis (Fig. 2*C*) and suggests that O_2_ is the preferred state adopted by unbound PDI in solution, regardless of the redox state. Other differences between the two redox states concerned the magnitude of the rate constants. We found that the rate at which O_2_ converts to C (k_2,3_) was approximately eight times faster for oxidized PDI than for reduced PDI. In contrast, the rate at which C converts to O_1_ (k_3,1_) was approximately nine times slower in oxidized PDI compared to reduced PDI. Faster O_2_→C conversion and slower C→O_1_ conversion explain why more C is present in oxidized PDI than in reduced PDI. We also found that the transition O_1_⟷O_2_ was the fastest of the catalytic cycle and significantly faster (approximately sixfold) than diffusion, especially for reduced PDI. This latter observation is important for two reasons. First, it explains why O_1_ and O_2_ cannot be individually visualized in the plots of FRET efficiency *versus* lifetime but instead merge to form a broad ensemble. Second, it predicts that when PDI dwells in O_1_ or O_2_, transition to C is energetically more expensive, especially when PDI is reduced. From a structural standpoint, this indicates that O_1_ and O_2_ may be alike yet significantly different from C.

To further confirm that O_1_ and O_2_ exchange rapidly, we performed species-selected filtered fluorescence correlation spectroscopy (fFCS). In this method, as described by Felekyan *et al*. (26), autocorrelation and crosscorrelation functions are calculated for two species of interest to determine the presence of dynamic exchange between them and the interconversion rates. fFCS analysis was performed between O_1_ (0.17 < E < 0.25) and O_2_ (0.65 < E < 0.75) in oxidized and reduced states. The results are shown in Figure 2, *F* and *G*. The presence of a bell-shaped crosscorrelation function between O_1_ and O_2_ documents rapid exchange occurring on a time scale comparable to or faster than the diffusion time. Global fitting of the four correlation curves (two sACFs and two sCCFs) required, in addition to the diffusion term (τ_D_), an additional relaxation term, τ_R_, providing conclusive evidence of fast dynamics. After fixing the diffusion term to 469 μs, the values of τ_R_ calculated for oxidized and reduced 88/467 were 119 ± 12 μs and 80 ± 9 μs, respectively. These values are in reasonable agreement with the rate constants measured by PDA for the O_1_⟷O_2_ exchange (τ_R_=(k_1,2_ +k_2,1_)^−1^), which are 145 μs for oxidized PDI and 67 μs for reduced PDI, respectively. Thus, fFCS successfully visualized rapid exchange between O_1_ and O_2_ and confirmed that one of the main differences between oxidized and reduced PDI is the ability of O_1_ and O_2_ to exchange faster in reduced PDI but slower in oxidized PDI.

### Role of the R300-W396 cation-π interaction

**Figure 3:**
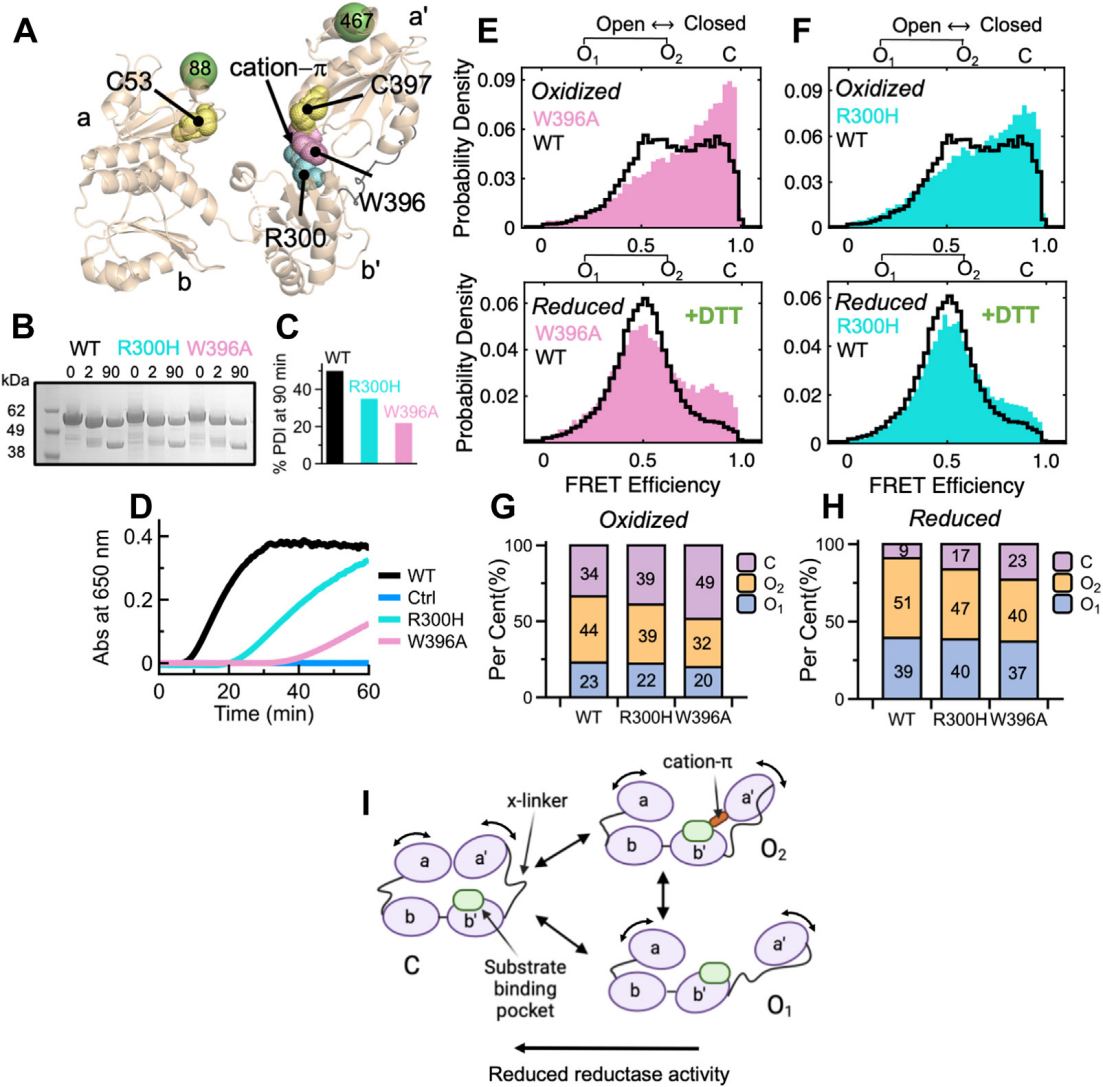
**Role of the PDI R300-W396 cation-π interaction.***A*, position of the R300-W396 cation-π interaction relative to the active sites and residues 88/467 captured in the X-ray crystal structure of reduced PDI. *B*, limited proteolysis of PDI by proteinase K monitored by SDS-PAGE. Samples were quenched at 0, 2, and 90 min. *C*, percent of intact PDI (MW 50 kDa) after 90 min of proteolysis. *D*, reductase activity of WT and variants monitored by the insulin assay. *E* and *F*, normalized FRET efficiency histograms of oxidized (*top panels*) reduced (*bottom panels*) W396A and R300H overlayed with WT (*black*) document a significant shift toward the closed ensemble. *G* and *H*, quantification of C, O_1_, and O_2_ obtained using PDA. *I*, conformational cycle of PDI highlighting structural differences between O_1_ and O_2_. Note how the major substrate-binding pocket (*green*) is likely to be more accessible in O_1_ and O_2_ but partly hidden in C due to compaction of the molecule. Also shown is the **x**-linker, which likely changes its conformation while transitioning between the ensembles. PDI, protein disulfide isomerase.

To ensure coordinated transitions between the ensembles, specific interdomain interactions must exist. An important interaction identified by X-ray crystallography (16) and corroborated by mutagenesis studies (27) is the cation-π interaction between residues R300 and W396 in the **b’** and **a’** domains, respectively (Fig. 3*A*). To test the contribution of this interaction, residue W396 was mutated to alanine (A) and R300 was mutated to histidine (H) since the mutation R300H predisposes to ALS (28). Previous studies have found that the mutants W396A and R300A are cleaved faster than WT by proteinase K, indicating that the **x**-linker is more accessible to the proteolytic enzyme (27). We verified this observation in our mutants after labeling (Fig. 3, *B* and *C*). We also found that the reductase activity of W396A and R300H toward insulin was significantly compromised compared to PDI 88/467 (referred to hereafter as WT) (Fig. 3*D*). Next, we performed smFRET measurements as described before. We found that the FRET profiles of both variants, and especially W396A, were shifted toward high FRET compared to oxidized and reduced WT (Figs. 3, *E* and *F* and S12). This indicates that the R300-W396 cation-π interaction stabilizes open conformations of PDI. PDA substantiated this observation and further revealed that accumulation of C arises from the loss of O_2_, whereas O_1_ was minimally affected (Fig. 3, *G* and *H*). Hence, these data validate the importance of the cation-π interaction in solution and suggest that one of the main structural differences between O_1_ and O_2_ is the absence of the R300-W396 cation-π interaction in O_1_ but its presence in O_2_ (Fig. 3*I*). Importantly, these data also indicate that modulation of PDI’s reductase activity occurs *via* redistribution of the structural ensembles, specifically loss of O_2_ and accumulation of C, thus linking structure to function.

### Role of the active site cysteines

**Figure 4:**
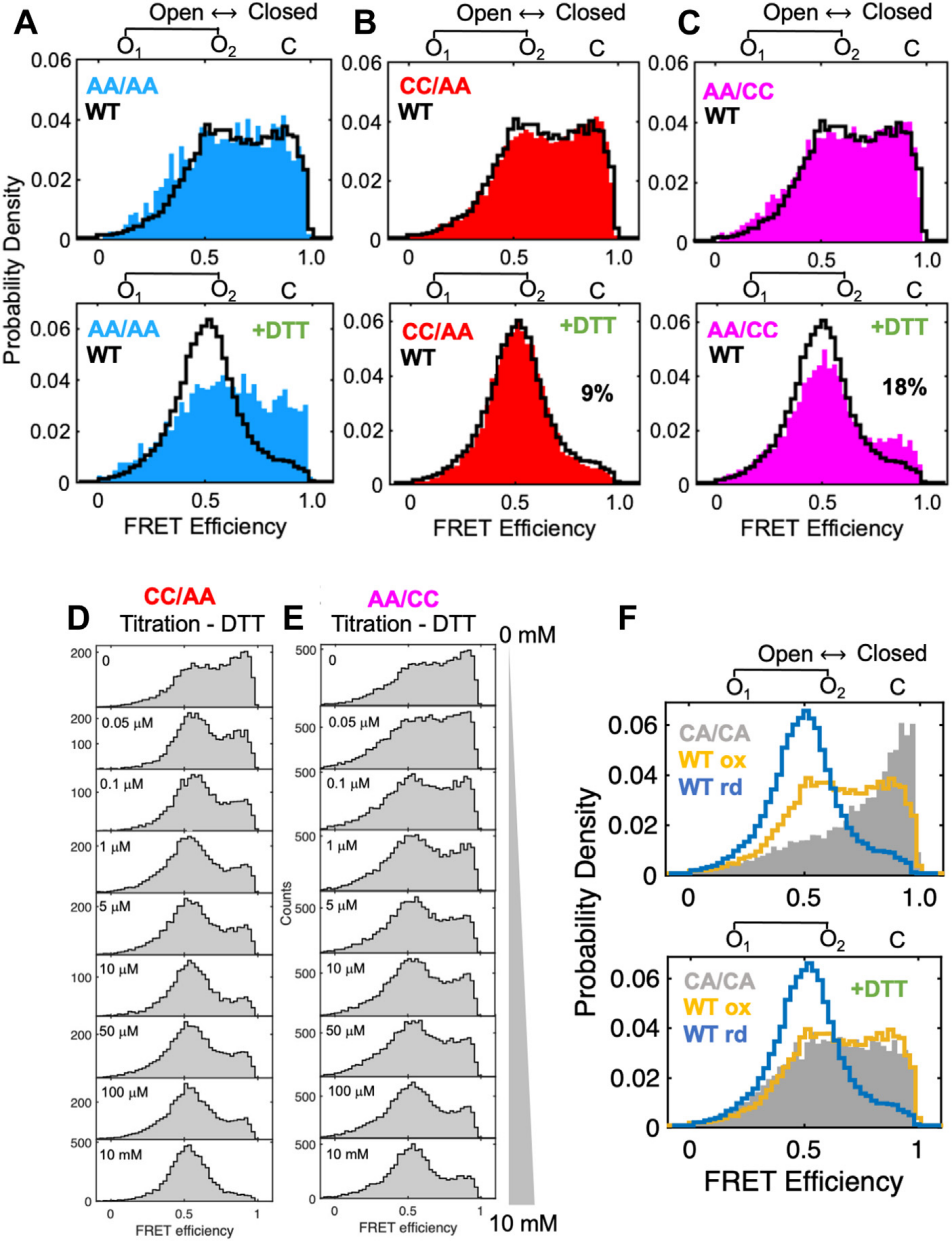
**Structural nonequivalence of the PDI active site cysteines.***A*–*C*, normalized FRET efficiency histograms of (*A*) AA/AA (*blue*), (*B*) CC/AA (*red*), and (*C*) AA/CC (*magenta*) overlaid to WT (*black*) before (*top panel*) and after (*bottom panel*) the addition of 1 mM DTT. Note how AA/AA is similar to oxidized PDI WT and insensitive to DTT and how the mutations C53A and C56A led to a macroscopic accumulation of C, which, according to PDA analysis (Table 1), increased two times, from 9% to 18%. *D* and *E*, FRET efficiency histograms of CC/AA (*D*) and AA/CC (*E*) were collected at increasing concentrations of DTT (0–10 mM), covering 4 orders of magnitudes. *F*, FRET efficiency histograms of CA/CA before (*top panel*) and after (*bottom panel*) the addition of 1 mM DTT overlayed to FRET distributions of oxidized (*yellow*) and reduced (*blue*) WT. PDI, protein disulfide isomerase.

PDI has four catalytic cysteines (C), two in the N-terminal **a** domain (*i.e.*, C53/C56) and two in the C-terminal **a’** domain (*i.e.*, C397/C400). It is believed that redox-dependent conformational changes in PDI are driven by disulfide bond formation in the **a’** domain (16) and that the minimum redox-regulated cassette is located in the **b’xa’** (27). According to this model, mutations of C to the redox-insensitive amino acid alanine (A) are predicted to stabilize PDI into the reduced state, and mutations of residues C397 and C400 to A should abrogate redox-dependent conformational changes in PDI. We generated AA/AA, CC/AA, and AA/CC in the 88/467 background to test these hypotheses. After verifying that the mutants have activity profiles consistent with what was previously reported in the literature for the WT background (29) (Fig. S13), smFRET measurements were collected in the absence and presence of DTT. PDA was used to quantify the species at equilibrium as described previously. Results of PDA are reported in Table 1. FRET efficiency plots comparing WT and mutants are shown in the main text (Fig. 4). FRET efficiency *versus* lifetime plots of the mutants are shown in Fig. S14.

Evaluating oxidized constructs, we found that AA/AA, CC/AA, and AA/CC were similar to each other and similar to oxidized WT. As expected, AA/AA was insensitive to DTT (Fig. 4*A*). These data indicate that replacing C with A results in an enzyme that more closely resembles oxidized and not reduced PDI. Since AA/AA is insensitive to DTT, they also provide additional and independent evidence that FRET changes observed with CC/CC are due to structural reorganization of the catalytic domains and not spectroscopic artifacts induced by DTT.

Next, we found that, although similar in the oxidized form, CC/AA and AA/CC behaved quite differently in the presence of DTT. While CC/AA mirrored the behavior of WT (Fig. 4*B*), AA/CC responded incompletely to the addition of DTT, leading to a significant twofold accumulation of C (Fig. 4*C*). Since high concentrations of DTT did not rescue the defect, accumulation of C must be due to changes in protein dynamics caused by the mutations rather than reduced reactivity of the mutant toward DTT (Fig. 4, *D* and *E*). We conclude that, differently from what was expected, the presence of reduced cysteines, rather than disulfide bonds, induces redistribution of the ensemble of conformations and that of the two active sites, the one in the N-terminal serves an important role in regulating PDI dynamics.

Of the four cysteine residues, two (*i.e.*, C53 and C397) attack the incoming substrate, whereas the other two (*i.e.*, C56 and C400) resolve the enzyme–substrate complex. Since the resolving cysteines are protonated at pH 7.4 but the attacking cysteines are not (3), they may play different structural roles. To identify which set of cysteines is responsible for the stabilization of reduced PDI in the open ensembles, we generated the variants C56A/C400A (CA/CA) and C53A/C397A (AC/AC) in the 88/467 background. We expected one of the two variants to be identical to reduced WT in the presence of DTT. While CA/CA expressed at a yield sufficient for labeling, AC/AC, unfortunately, did not. Nonetheless, data with CA/CA were extremely informative. Based on previous studies (17), we expected to measure high FRET for oxidized CA/CA due to the formation of a disulfide bond between C53 and C397 and lower FRET in the presence of DTT due to cleavage of the disulfide bond. Consistent with these predictions, we found that after purification, CA/CA mostly populates C (∼80%) and that the addition of DTT resulted in lower FRET (Fig. 4*F*). Reduced CA/CA was, however, similar to oxidized, not reduced WT, thus suggesting that the resolving cysteines are crucial for enabling reduced PDI to reside in open ensembles.

### Active site ligation stabilizes closed conformations of PDI

**Figure 5:**
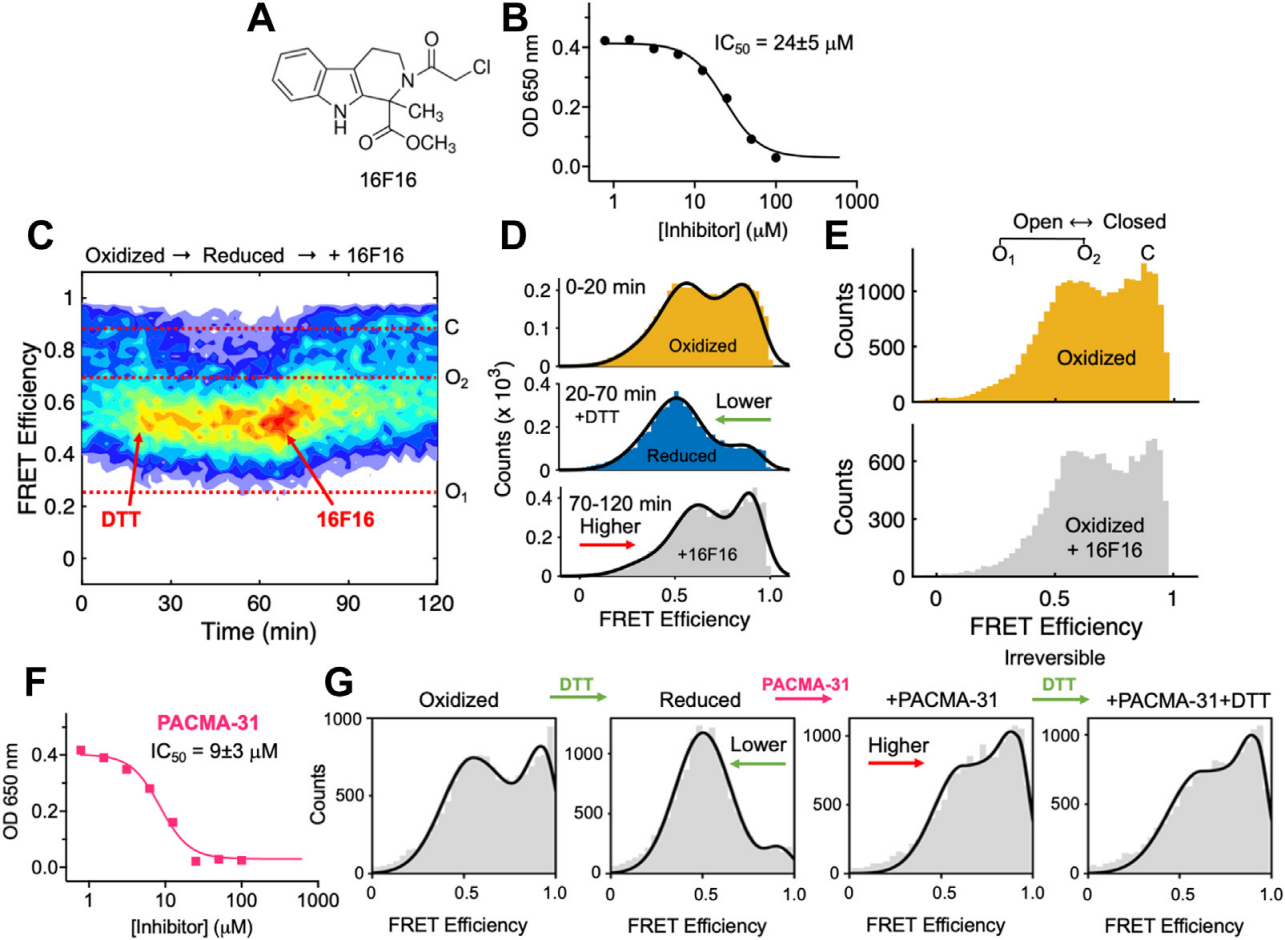
**Active site ligation stabilizes closed conformations of PDI.***A*, chemical structure of 16F16. *B*, inhibition of PDI’s reductase activity by 16F16 measured using the insulin assay and IC_50_ value. *C*, ligand binding followed by smFRET. A solution of PDI 88/467 (50 pM) was continuously monitored for 120 min under different experimental conditions. Addition of 50 μM DTT and 50 μM 16F16 is indicated with *red arrows*. The horizontal dotted lines identify the mean FRET efficiency value of C, O_1_, and O_2_. *D*, FRET efficiency histograms of the reaction of WT with 16F16 at three different time intervals. *E*, lack of reactivity of 16F16 toward oxidized WT. *F*, inhibition of PDI’s reductase activity by PACMA-31 measured using the insulin assay and IC_50_ value. *G*, FRET histograms of WT in the absence and presence of DTT (1 mM) before and after adding 50 μM PACMA-31. Note how PACMA-31, like 16F16, shifts the conformational equilibrium toward the closed ensemble. Further addition of DTT is inconsequential. This is because PACMA-31 reacts irreversibly with the active site thiol groups of PDI to form a covalent adduct. PDI, protein disulfide isomerase.

The active site thiols of PDI are among the most reactive in the human proteome (30) and can react with a variety of electrophiles. Over the years, several compounds targeting these cysteines have been developed and used as irreversible inhibitors by multiple investigators in various studies (9, 31). Of those, 16F16 (Fig. 5*A*) is one the most common and potent (Fig. 5*B*) (32). Yet, how 16F16 affects the conformation of PDI remains unknown. Since 16F16 does not quench the fluorescence intensity of the Atto dyes, the conformational changes resulting from their interaction with PDI were monitored in real-time by smFRET. The addition of 16F16 to reduced PDI did not abolish protein dynamics but shifted the equilibrium toward C in a time-dependent fashion (Fig. 5, *C* and *D*). In contrast, 16F16 did not change the FRET profile of oxidized PDI (Fig. 5*E*), confirming the selectivity of the compound toward the reduced enzyme and ruling out conformational effects induced by binding of 16F16 to the adjacent **b** and **b’** domains that might occur before interacting with the active site thiols. Similar results were obtained with PACMA-31 (Fig. 5, *F* and *G*), which, like 16F16, irreversibly inhibits PDI (33). This suggests that compaction of PDI may be a general feature for this class of compounds.

## Discussion

PDI is an enzyme essential for life and is involved in several human disorders. Hence, it has been studied for decades. Here, we used multiparameter confocal smFRET to investigate the conformational dynamics of PDI in solution. This approach demonstrated several unexpected new features of this enzyme. PDI comprises four thioredoxin domains organized to form a flexible **abb’a’** U-shape. To track movements of the catalytic domains, we designed four FRET pairs based on the only two available crystal structures of unbound oxidized and reduced PDI. We expected higher FRET in the presence of a reducing agent, such as DTT. However, we systematically observed lower FRET upon reduction. This indicates that the crystal structures of PDI are not the lowest energy conformers in solution and that, upon reduction, PDI is more open than oxidized PDI—where open indicates a greater distance between the two catalytic domains. We also found that PDI often populates conformers in which residues 57/401 and residues 88/401 are closer than ∼45 Å. This is because the R_0_ for the FRET pair Atto-550/647N is 62.3 Å; hence, the sensitive detection range is limited to 4 to 9 nm. A possibility well supported by computational studies (10, 17, 34) is that PDI undergoes hinge-bending movements and rotations of the **a’** domain relative to **a** domain in a controlled fashion so that the active sites face, most of the time, the inner part of the molecule, which is where the substrates bind. Proximity and proper orientation of the active sites could facilitate PDI operations *via* cooperativity, a concept proposed years ago for yeast PDI (35). We have previously shown that residues 42 and 467 in the **a** and **a’** domains and 308 and 467 in domains **b’** and **a’** become further apart upon reduction with DTT (12). The findings reported here documenting opening of PDI upon reduction are consistent with our previous findings, and together, they establish that, in solution, movements of the catalytic domains are facilitated by the flexibility of the **x**-linker.

In addition to documenting significant distance changes between **a** and **a’**, we showed that reduced PDI is structurally less heterogeneous than oxidized PDI even though it is more open. This is because, unlike oxidized PDI, reduced PDI rapidly exchanges between O_1_ and O_2_ and rarely visits C. As a result, the FRET profiles of 88/467 and 57/467 narrowed in the presence of DTT. Okumura *et al*. (18) also reported narrower distributions for reduced PDI using HS-AFM, which, unlike what we found, appeared to be more compact and less dynamic than oxidized PDI. The reason for such a discrepancy is currently unknown. Still, it might be due to the lower time resolution of HS-AFM compared to confocal smFRET and the fact that both techniques, unfortunately, provide low-resolution structural information. Furthermore, we studied PDI in solution rather than PDI fixed to mica sheets. Immobilization of flexible proteins onto surfaces might alter protein dynamics to a greater extent than site-specific incorporation of fluorescent dyes. Hence, while conceptually similar in that both studies found that the structural flexibility of PDI is modulated by the redox environment, the results of our studies suggest that reduced PDI is open and flexible, not compact and rigid. Redox-dependent conformational changes were also studied by small-angle X-ray scattering (SAXS) (18), which is a solution technique providing low-resolution structural information. The authors found that the SAXS profiles of oxidized and reduced PDI disagree significantly with those calculated from their crystal structures, indicating that PDI adopts different conformations in solution. This observation agrees with our findings. However, the authors also reported that oxidized PDI assumes a slightly more extended conformation than reduced PDI, a conclusion that is not recapitulated by our smFRET data. SAXS measurements require concentrations of protein ∼3 orders of magnitude higher than the concentrations used for smFRET experiments. Interestingly, at these concentrations, the same study shows that PDI forms dimers (18). Because PDI forms dimers under experimental conditions used to collect SAXS data, differences in the SAXS profiles are more complicated to interpret in terms of structural compaction of the monomeric form since they could reflect differences in the monomer–dimer equilibrium. Future studies using orthogonal techniques are required to address these discrepancies.

**Figure 6:**
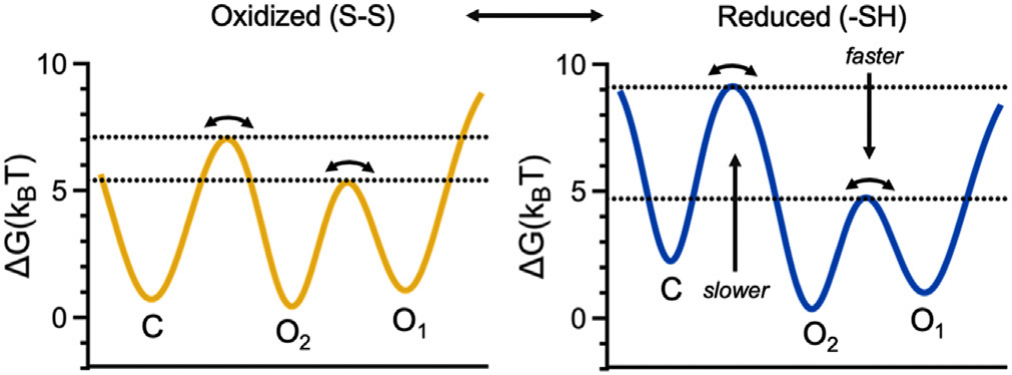
**Conformational landscape of PDI inferred by smFRET.** Free energy profiles of oxidized (left, *yellow*) and reduced (right, *blue*) PDI obtained using a linear scheme and data reported in Table 1. Barrier heights corresponding to C→O_2_ and O_1_→O_2_ (horizontal lines) for oxidized and reduced PDI were calculated using the Kramers equation with a pre-exponential factor of 10^5^ s^−1^. Even though the best empirical estimates for protein dynamics are around 10^6^ s^−1^, this value was chosen based on previous studies to facilitate comparison with other protein systems (13, 36). k_B_ is the Boltzmann constant, and T is the temperature. The distributions were arbitrarily drawn using a combination of Gaussian distributions. Note how in the presence of DTT, the free-energy barrier increases for the C⟷O_1_/O_2_ transition (slower transition), whereas it decreases for the O_1_⟷O_2_ transition (faster transition), leading to redistribution of the conformational ensembles. PDI, protein disulfide isomerase; smFRET, single-molecule FRET.

Even though several studies concluded that PDI is a remarkably flexible enzyme, none of them addressed how fast the domains move in solution. The high temporal resolution of our confocal instrument enabled us to measure the rate constants at which the ensembles interconvert, hence documenting that the **a** and **a’** domains move remarkably fast, in the submillisecond timescale. This suggests that domain motions, in addition to controlling substrate binding and product release, may also contribute to fine-tuning the nucleophilicity of the active sites. Importantly, the availability of the reaction rate constants reported in Table 1 also allowed us to calculate free-energy barriers for transition between the ensembles, hence allowing visualization of the free-energy landscape of PDI (Fig. 6). For both oxidized and reduced PDI, we found low basins, shallow minima, and transitions between ensembles characterized by Gibbs free-energy values (ΔG) lower than 10K_B_T, which is remarkably low. Since 6.7K_B_T is the energy required to form/break one hydrogen bond (36), this analysis indicates that the transition between the ensembles requires formation/breakage of less than two hydrogen bonds, explaining why PDI is so conformationally flexible. Since the energy to form/break a cation-π interaction accounts for 3-9K_B_T (37), this analysis also effectively explains why the single point mutations W396A and R300H dramatically affect the conformational landscape of PDI.

There has been much interest in how the catalytic motif CxxC controls PDI dynamics. The most popular view is that oxidation of catalytic cysteines in the **a’** domain, hence formation of a disulfide bond, results in significant conformational changes sensed by the **x**-linker, leading to more open conformations of the enzyme (27). In contrast to this model, here we show that the variant AA/AA, which is incapable of forming disulfide bonds, can adopt closed and open ensembles with similar probability. These results indicate that the AA/AA variant is similar to oxidized, not reduced, PDI. We also found that CC/AA, but not AA/CC, was identical to WT. Hence, the N-terminal active site has a major regulatory role in PDI dynamics. A critical role for the N-terminal catalytic domain in controlling PDI conformation has not previously been observed and challenges the dogma that the minimum redox-regulated cassette is located in the **b’xa’** (27). Finally, we found that CA/CA in the presence of DTT resembles oxidized WT. Combined with AA/AA, CC/AA, and AA/CC, results with the variant CA/CA indicate that formation of active site thiols in the **a** domain is the mechanism enabling reduced PDI to reside in open ensembles and that residue C56 is likely to play a key role in this process.

Previous studies have suggested that oxidized and reduced PDI are structurally different (16). Based on a recent crystal structure of reduced PDI bound to the alpha subunit of microsomal triglyceride transfer protein (19) and molecular dynamics simulations (10, 17), others have proposed that PDI has similar conformational flexibility in both states that is needed for substrate binding. By documenting movements of the catalytic domains that are stimulated, yet not initiated, by various factors such as the redox environment, single point mutations, and inhibitor binding, our FRET data provide convincing support to the second hypothesis. Specifically, we propose that both oxidized and reduced states of PDI are flexible and rapidly oscillate between conformations that are distinct from each other but shared by both redox states. Hence, thinking of oxidized PDI as an open and flexible conformation and reduced PDI as a close and rigid conformation is inconsistent with our data and too simplistic for rationalizing the conformational cycle of this extremely flexible enzyme. Instead, selection between preexisting ensembles, perhaps followed by additional conformational changes, provides the structural basis for understanding how PDI activity is regulated *via* conformational modulation *in vivo.* For example, it is well established that both reduced and oxidized PDI can recognize substrates. Indeed, the ability for both oxidized and reduced PDI to recognize the same substrate is essential to its ability to act as an isomerase. Its activity as an oxidoreductase may also derive from the fact that both oxidation states visit similar conformations, albeit to different extents. On these bases, we propose that rapid sampling of multiple conformations contributes to the ability of PDI to recognize multiple substrates. Enhanced ability to recognize multiple substrates resulting from its dynamic nature would also improve its capacity as a chaperone.

## Experimental procedures

### Protein production and purification

The complementary DNA of human PDI (residues 18–479) was cloned into a pBAD vector expression system (ThermoFisher) and modified to include an N-terminal 6 His-tag and a C-terminal Avitag. Genetic incorporation of the unnatural amino acidic N-Propargyl-L-Lysine (Prk) (SiChem) at positions 57, 88, 401, and 467 was obtained using the AMBER suppressor pyrrolysine tRNA/RS system from *Methanosarcina mazei*. Mutations C53A, C56A, C397A, C400A, W396A, and R300H in the 88/467 background were generated using the Quickchange Lightning kit (Agilent) with appropriate primers. Sequence verified PDI variants (Genewiz) were expressed in Top10 cells and purified following recently published procedures (12).

### Protein labeling

Site-specific labeling was achieved as detailed elsewhere (12). Briefly, a solution of 25 μM of PDI in 1x PBS pH 7.4 (Corning) was reacted with 4x molar excess of azide dyes (donor, acceptor, or donor/acceptor mixtures) (Sigma–Aldrich) in the presence of 150 μM copper sulfate (CuSO_4_), 750 μM tris-hydroxypropyltriazolylmethylamine, and 5 mM sodium ascorbate. The reaction mixer was left on a slow rotisserie for 1 h 30 min at room temperature (RT), then 30 min on ice. The reactions were stopped by adding 5 mM EDTA. Monomeric PDI was successfully separated by protein aggregates by size-exclusion chromatography, using a Superdex 200 10/300 column (Cytiva) equilibrated with Tris 20 mM (pH 7.4), 150 mM NaCl, and 2 mM EDTA. The quality of each protein preparation was assessed by NuPAGE Novex 4 to 12% Bis–Tris protein gels (ThermoFisher). Gels were stained with Coomassie brilliant blue R-250 (ThermoFisher) and scanned on a Typhoon imager (Cytiva) at 532 nm and 633 nm to verify specific incorporation of the fluorescent dyes. Total protein concentration was determined by reading the absorbance at 280, using a molar coefficient adjusted for the amino acidic sequence of each variant. The concentration of Atto-550 and Atto-647N was calculated by reading the absorbance at 554 nm and 636 nm, respectively. Correction factors at 280 nm for Atto-550 and Atto-647N were 0.1 and 0.03, respectively. Typical labeling efficiencies for the variants reported in this study were ∼50% (Fig. S1).

### Ellman’s reagent assay

Ellman’s reagent (DTNB, 5,5′-dithiobis(2-nitrobenzoic acid)) (ThermoFisher) was dissolved in 100 mM sodium phosphate, 1 mM EDTA, pH 8.0 (PBS) in the absence or presence of 3M guanidium chloride (Gnd-HCl) at 4 mg/ml and subsequently diluted to 0.1 mg/ml. To determine free thiol concentration, 5 μl of 37.5 μM of doubly labeled PDI 88/467 was mixed with 10 μl of DTNB in a PCR tube and incubated for 20 min at RT in the dark. In this reaction, the thiolate form of the cysteine sulfhydryl group reacts with Ellman's reagent to form 2-nitro-5-thiobenzoate anion (TNB^2−^), which absorbs light at 412 nm, A_412_. Spectra were recorded using a Nanodrop One (ThermoFisher) allowing quantification of TNB^2−^. The concentration of TNB^2−^ is calculated after subtraction of baseline using the Lambert–Beer law using a molar extinction coefficient of 11,400 M^−1^ cm^− 1^ (38). A standard curve was prepared using cysteine hydrochloride monohydrate (0–62 μM).

### CD

Far-UV CD spectra were recorded on Jasco J-715 spectropolarimeter equipped with a water-jacketed cell holder, connected to a water-circulating bath, as done before (12). Spectra were collected for unlabeled and labeled protein in PBS with 2 mM EDTA at a concentration of 0.12 mg/ml. The final spectra resulted from the average of five accumulations after baseline subtraction. Data were analyzed using Origin 7.5 (OriginLab).

### Intrinsic fluorescence assay

Intrinsic fluorescence spectra (tryptophan) were performed in a reaction volume of 200 μl with 0.2 μM of PDI in 20 mM Tris–HCl buffer containing 150 mM NaCl (pH 7.4) and either 1 mM GSH or 1 mM GSSG were incubated for 1 h at RT. Emission spectra were recorded at 295 to 450 nm with excitation at 280 nm using a FluoroMax-4 (Horiba). Data were analyzed using Origin 7.5.

### Insulin reductase assay

PDIs (400 nM) were solubilized in PBS pH 7.4 and then added to a solution containing 0.2 mM human insulin (Sigma–Aldrich), 2 mM EDTA, and 325 μM DTT. The reaction was monitored at 650 nm (turbidity due to precipitation of the product) for 1 h at 25  °C using a Spark multimode plate reader (Tecan). Increasing concentrations (0–100 μM) of the inhibitors 16F16 (Sigma–Aldrich) and PACMA-31 (Sigma–Aldrich) were incubated for 10 min with PDI before adding insulin. Data were analyzed using Origin 7.5 and graphed using Prism 9.0 (GraphPad Software).

### Digestion with proteinase K

PDI 88/467 and mutants R300H and W396A were dissolved in 20 mM Tris, 150 mM NaCl, 2 mM EDTA, pH 7.4, and 1 mM DTT at 1 mg/ml (60 μl). Following the addition of proteinase K (Sigma–Aldrich) (final concentration 5 μg/ml), aliquots (16 μl) were quenched at different time intervals with 1 mM PMSF (2 μl) for 2 min at RT before flash freezing. The quenched reaction was then mixed with 30 μl of NuPAGE LDS buffer and heated at 95 °C for 10 min. Samples were loaded into NuPAGE Novex 4 to 12% Bis–Tris protein gels (ThermoFisher) run with MES buffer. Gels were stained with Coomassie brilliant blue R-250 (ThermoFisher) and analyzed by quantitative densitometry using ImageJ (https://imagej.nih.gov/ij/index.html).

### Determination of anisotropy and quantum yield

Four singly labeled PDI constructs (*i.e.*, K57U, S88U, K401U, and K467U) were expressed, labeled with either Atto-550 or Atto-647N, and purified as described before for anisotropy and quantum yield determination. Anisotropy was recorded in 1x PBS with 2 mM EDTA buffer using Fluorolog-3 (Jobin Yvon). The Atto-550 labeled proteins (50 nM) were excited at 540 nm and emission was monitored at 580 nm. The Atto-647N labeled proteins (50 nM) were excited at 640 nm and emission was monitored at 680 nm; excitation and emission slits were set at 1 and 14 nm, respectively. The donor quantum yield was measured in bulk fluorescence assays in a FluoroMax-4 (Jobin Yvon) for each donor position, in reference to the quantum yield of rhodamine 110 (89.87 ± 0.91). The emission spectra of PDI labeled with only a donor at positions 57, 88, 401, and 467 were collected at five concentrations under the same excitation conditions (532 nm) in the buffer used for smFRET experiments. The quantum yield was found from the ratio between the dye’s integrated emission spectrum and its absorbance at 532 nm. The overlap between the donor emission spectrum and the acceptor absorbance spectrum is defined as:

$$J\left(\lambda \right)=\overset{\infty }{\underset{0}{\int }}F_{D}\left(\lambda \right)\varepsilon_{A}\left(\lambda \right)\lambda^{4}\text{d}\lambda$$

where F_D_ (l) is the normalized donor emission spectrum and e_A_ is the acceptor’s absorbance spectrum, measured for PDI labeled with an acceptor at positions 57, 88, 401, and 467, respectively.

### smFRET measurements

FRET measurements of freely diffusing single molecules were performed with a confocal microscope MicroTime 200 (PicoQuant) using published procedures (39, 40). Excitation laser light from 532 nm and 638 nm lasers was used to excite the donor and acceptor fluorophores, respectively. A pulsed interleaved excitation setup was used with a pulse rate of 20 MHz to alternate the donor and acceptor excitation. Pulsed interleaved excitation reports the status of both donor and acceptor fluorophores by sorting molecules based on relative donor:acceptor stoichiometry (S) and apparent FRET efficiency (E), as described before (14, 22, 41, 42). A dichroic mirror (ZT405/488/532/640rpc-XT, Chroma) reflecting at 532 and 638 nm guided the light to a high numerical aperture apochromatic objective (60x, numerical aperture 1.2, water immersion, Olympus) that focused the light to a confocal volume of 1.0 fl. Fluorescence from excited molecules was collected with the same objective and focused onto a 50 μm diameter pinhole. The donor and acceptor emissions were separated *via* a dichroic filter with a dividing edge at 620 nm (620DCXR, Chroma). Suited bandpass filters (HQ580/70m, Chroma and HQ690/70m, Chroma) were inserted to eliminate the respective excitation wavelength and minimize spectral crosstalk. The fluorescence was detected with two single-photon avalanche diode detectors (t-SPAD, PerkinElmer) using time-correlated single photon counting (TCSPC) with the TimeHarp 200 board (HydraHarp 400, PicoQuant). Data were stored in the time-tagged time-resolved mode as a PTU file format.

Measurements were performed 25 μm deep in the solution using a laser power of ∼15 μW. Total acquisition time was ∼40 min per sample. Data collection was repeated a minimum of four times using the same sample as well as new protein samples from at least one different preparation. Identical results were obtained in all cases, indicating stability of the protein sample during data collection and reproducibility. Concentration was 50 to 100 pM of labeled protein solubilized in 200 μl of 20 mM Tris, 150 mM NaCl, 2 mM EDTA, 0.003% Tween 20, pH 7.4 (TBSE-T) for oxidized PDI and TBSE-T with 1 mM DTT for reduced PDI. Tween 20 0.003% was used to minimize nonspecific absorption to the imaging chamber and, at this concertation, does not affect protein conformational dynamics as shown before (12). DTT titrations were performed by preincubating oxidized PDI with the desired concentration of DTT for 40 min at RT before data collection to ensure equilibrium. However, similar results were obtained by adding increasing concentrations of DTT to the same protein sample, implying fast reactivity of DTT toward the enzyme. The PDI inhibitors 16F16 and PACMA-31 solubilized in dimethyl sulfoxide were added to the solution at a final concentration of 50 μM (0.25% final dimethyl sulfoxide). Data recording was performed using the SymPhoTime Software 64, version 2.4 (PicoQuant).

### smFRET analysis

Data analysis was carried out with the Matlab-based software PAM (https://pam.readthedocs.io/en/latest/) (43) using a customized profile optimized for our microscope. Signals from single molecules were observed as bursts of fluorescence. Bursts with more than 40 counts were searched with the All Photon Burst Search (APBS) algorithm. Integration time was set to 0.5 ms. Appropriate corrections for direct excitation of the acceptor at the donor excitation wavelength (DE), leakage of the donor in the acceptor channel (Lk), and the instrumental factor (g) were determined experimentally using a mixture of dsDNA models with known FRET efficiency and stoichiometry labeled with dyes Atto-550 and Atto-647N (Fig. S9). These are the following: DE=0.05, Lk=0.08, g = 0.85.

A plot of the stoichiometry *versus* the ALEX-2CDE filter was used to determine the required upper threshold that removes donor-only (S = 1) and acceptor-only (S = 0) molecules. In general, only molecules within the range S = 0.25 to 0.75 were considered in the final analysis. Doubly labeled photobleached molecules were further eliminated using the ALEX-2CDE (<14) and |TDX-TAA| (<0.5) filters as described before by Tomov *et al*. (44) and Kudryavtsev *et al*. (14), respectively. These stringent filters guarantee elimination of unwanted signal, as described before (14).

Lifetime was calculated using the lifetime module built in PAM after correction for instrument response factor. For the whole dataset, a double exponential decay function was used for the donor channel, whereas a single exponential decay function was used for the acceptor channel (43). Static and dynamic FRET lines were generated using PAM following previously published methods (14, 22, 25) and using an apparent linker length of 5 Å. Lifetime of donor in the absence of acceptor was determined using D only population, defined as 0.75 < S < 1. Methods and equations have been published before (22, 43) and are also described at https://pam.readthedocs.io/en/latest/.

Subpopulation-specific fluorescence lifetime analysis was also performed using PAM. However, for this type of analysis, the region of interest was first selected in the BurstBrowser module. Data were then systematically fit with one, two, and three exponential functions to identify the best fit. Decisions were made based on analysis of the weighted residuals. Donor-only and acceptor-only species were also selected to verify that, in contrast to the FRET interval belonging to the open ensemble, they satisfactory fit a single exponential decay. Methods and equations have been published before (22, 43) and are also described at https://pam.readthedocs.io/en/latest/.

Theoretical FRET values were obtained by coarse-grained simulations using the FRET-restrained positioning and screening (FPS) software (https://www.mpc.hhu.de/en/software/fps) (45). The dye dimensions were estimated to be 7.8, 4.5, and 1.5 Ǻ for Atto-550 and 7.15, 4.5, and 1.5 Ǻ for Atto-647N after minimization of their chemical structure using Maestro (Schrödinger). The linker lengths and widths used were 18 and 4.5 Ǻ for both dyes. After performing accessible volume simulations, the corresponding mean transfer efficiency was calculated by assuming rapid fluctuations of the interdye distance occurring on time scales similar to the fluorescence lifetime of the donor (∼3.2 ns in the absence of the acceptor). This assumption is justified based on the anisotropy values reported in Table S1 obtained for the labeled proteins.

### Dynamic PDA

PDA analysis was performed using the PDAfit module built in PAM. Proximity histograms were reconstructed by binning the same dataset at 0.25, 0.5, 0.75, and 1 ms. Histogram library with a grid resolution for E = 100 and a minimum number of photons of 10 per bin was chosen. The datasets were then fit using a dynamic three-state model. Distances calculated from lifetime analysis were fixed. The width of the distance distribution was also fixed at sigma = 0.045. This was determined from the measurement of several static double-stranded doubly labeled DNA (Fig. S9). The value of 0.045 also agrees with the results of recent studies aimed at comparing the accuracy and reproducibility of smFRET data among multiple laboratories (46). R_0_ = 62.3 Å for PDI 88/467 was determined experimentally using n = 1.4, k^2^=2/3, and quantum yield reported in Table S1. To assess robustness of the fit, PDA was repeated by systematically varying the initial value of the rate constants to 1, 0.5, and 0.75 ms^−1^ (min 0, max 10) while keeping the other settings identical. The results in Table 1 represent the average of these three independent determinations.

### Species selected fFCS

Data were collected using the same setup described before in which we added two SPAD detectors (Excelitas Technologies) for a total of two parallel and two perpendicular detectors and increased the laser power to ∼30 μW. Briefly, the light was simultaneously split (50:50) and rotated by a polarizing cube. While the configuration of the perpendicular path is described before, the configuration of the parallel path is the following: dichroic filter ZT633rdc-UF1 (Chroma), donor emission filter ET585/65m (Chroma), and acceptor emission filter ET700/75m (Chroma). Fine-tuning of the system was performed such as very similar fluorescent intensity values (5% difference) were obtained for the two sets of detectors. FRET efficiency histograms and values of lifetime obtained for the 2- and 4-SPADs setups were identical.

Species selected fFCS analysis was done using BurstBrowser module from PAM software. Microtime patterns for O_1_ and O_2_ states were obtained using FRET efficiency thresholds around the mean lifetime values obtained for O_1_ (E = 0.17–0.25) and O_2_ (E = 0.65–0.75) states. Signals from selected FRET efficiency regions were cross correlated after generating the appropriate TCSPC filters for the parallel and perpendicular channels. This eliminates the dead-time of TCSPC hardware and SPAD detectors. Four correlations functions, two autocorrelation functions, and two crosscorrelation functions between the species O_1_ and O_2_ were generated. The four curves were globally fitted using a single component diffusion and single exponential kinetic term, as described by Felekyan *et al*. (26), and letting the amplitude for crosscorrelation assume negative values. fFCS fit was carried out in FCSfit module from PAM. The diffusion time was fixed to 469 μs. This value was obtained from independent FCS measurements of reduced and oxidized PDI molecules at nanomolar concentrations. The ratio of the axial and lateral size of the confocal volumes were globally fixed, ρ = 4.6. This was obtained from independent FCS measurements using singly labeled calibration samples such as dsDNA or singly labeled PDI molecules at nanomolar concentrations. Capabilities of fFCS in our system were tested using Holliday Junctions, as described elsewhere (26). Methods and equations have been published before (22, 43) and are also described at https://pam.readthedocs.io/en/latest/.

## Data availability

Raw.ptu files (time-correlated single-photon counting data) from our single-molecule FRET experiments data have been deposited in a publicly accessible database, Zenodo (https://doi.org/10.5281/zenodo.6324223).

## Supporting information

This article contains supporting information (12, 16, 27, 38, 43).

## Conflict of interest

The authors declare that they have no conflicts of interest with the contents of this article.

## Supporting information

2022_R2_JBC_SI final
