# Supplementary material for: Reduction of protein disulfide isomerase results in open conformations and stimulates dynamic exchange between structural ensembles
Source: J Biol Chem. 2022 Jun 30;298(8):102217. doi: 10.1016/j.jbc.2022.102217 (PMC9352907; doi:10.1016/j.jbc.2022.102217)
Supplement: 2022_R2_JBC_SI final [file mmc1.docx]

Supplementary Information for

**Reduction of protein disulfide isomerase results in open conformations and stimulates dynamic exchange between structural ensembles**

Mathivanan Chinnaraj^1^, Robert Flaumenhaft^2^ and Nicola Pozzi^1,*^

*Corresponding authors:

Nicola Pozzi

Email: [nicola.pozzi@health.slu.edu](mailto:nicola.pozzi@health.slu.edu)

Phone: +1-314-977-9241

Robert Flaumenhaft

Email: [rflaumen@bidmc.harvard.edu](mailto:rflaumen@bidmc.harvard.edu)

Phone: +1-617-735-4005

**This PDF file includes:**

Figs. S1 to S14

Tables S1 to S2

References (1 to 5)

**
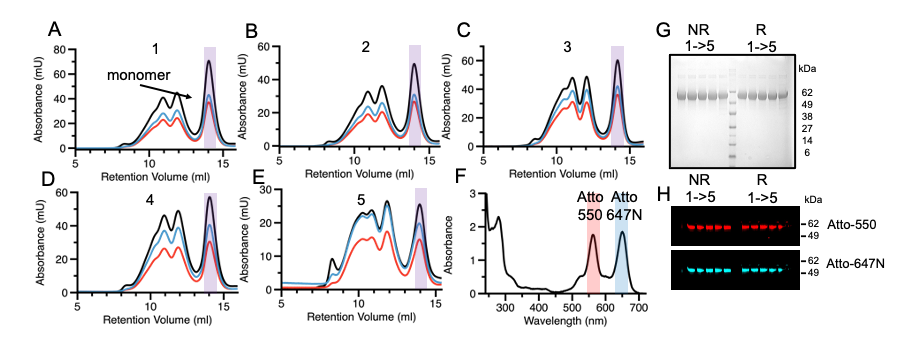
Figure S1. Purification and chemical characterization of PDI after incorporation of Atto dyes by click chemistry. A-E)** SEC chromatographic profiles of 88/467 after labeling. Highlighted in magenta is monomeric PDI, which elutes at ~14 ml. Reproducibility was proven by performing the same reaction 5 times using 3 different batches of protein and 3 different lots of dyes. Similar chromatographic profiles were obtained for all the variants used in this study. **F)** Representative UV-Vis spectrum of sample 1 after concentration using a 30 kDa cut-off filter. Sample 1 (35 μM) contains equal concentrations of Atto550 (11.6 μM) and Atto647N (12 μM) accounting for ~34% labeling efficiency. **G)** 4-12% SDS-PAGE of samples 1-5 run under non-reducing (NR) and reducing (R) conditions showing excepted MW and purity >95%. **H)** The same samples of figure G were diluted 20-fold, loaded in a new gel and imaged using fluorescence to verify the covalent incorporation of the dyes.

**
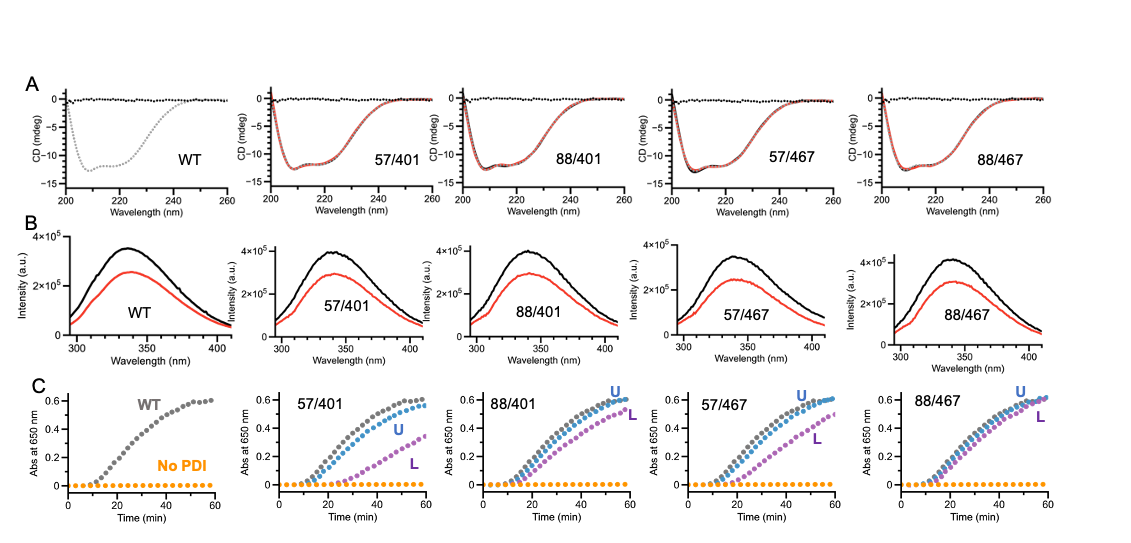
Figure S2. Structural and functional characterization of the FRET variants. A)** Far-UV CD spectra of doubly labeled 57/401, 57/467, 88/401, and 88/467 (red) show similar secondary structure compared to unlabeled proteins (black) and PDI WT (dotted lines). **B)** Response to GSSG (red) and GSH (black) monitored by intrinsic fluorescence. Doubly labeled 57/401, 57/467, 88/401, and 88/467 responded well to redox stimulation, as described before for PDI WT (1). **C)** Reductase activity monitored by the insulin assay. The labels U and L indicate unlabeled and doubly labeled protein samples, respectively. PDI WT is in gray. Yellow dots are control experiments containing insulin and DTT without PDI.


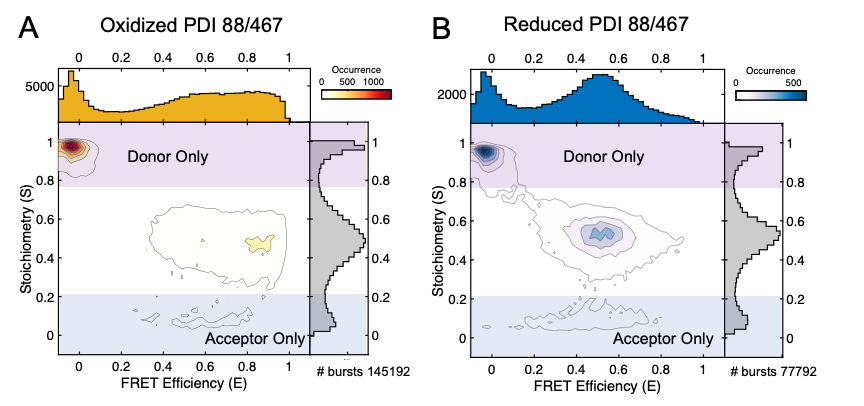
**Figure S3.** **FRET efficiency vs stoichiometry plot of PDI 88/467 before cleanup.** FRET efficiency vs stoichiometry plot of oxidized **(A)** reduced 88/467 **(B)** before cleanup. Highlighted are Donor only (S>0.75, magenta) and Acceptor only (S<0.25, blue) populations. These species were discarded as they are not relevant to our analysis, as described elsewhere (2,3).


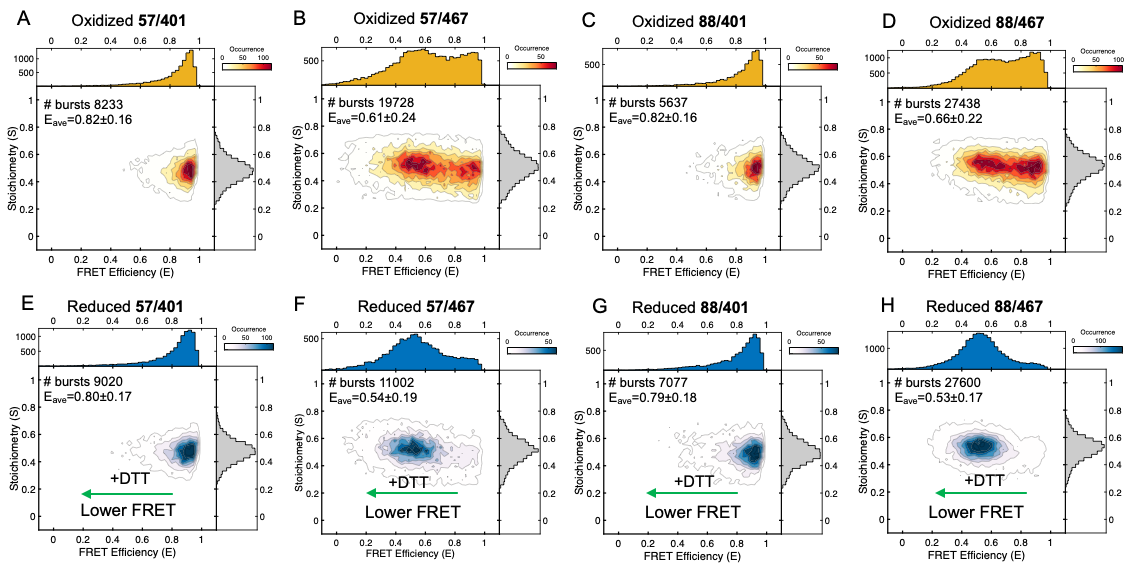


**Figure S4. FRET efficiency vs stoichiometry plots of PDI variants labeled with Atto dyes.** FRET efficiency vs stoichiometry plots of 57/401 **(A,E)**, 57/467 **(B,F)**, 88/401 **(C,G)** and 88/467 **(D,H)** under non-reducing (top, yellow) and reducing conditions (bottom, blue). Note how the addition of DTT, while shifting E_ave_ towards lower FRET (green arrow line), does not affect stoichiometry, consistent with a monomeric enzyme undergoing redox-stimulated conformational changes.

**
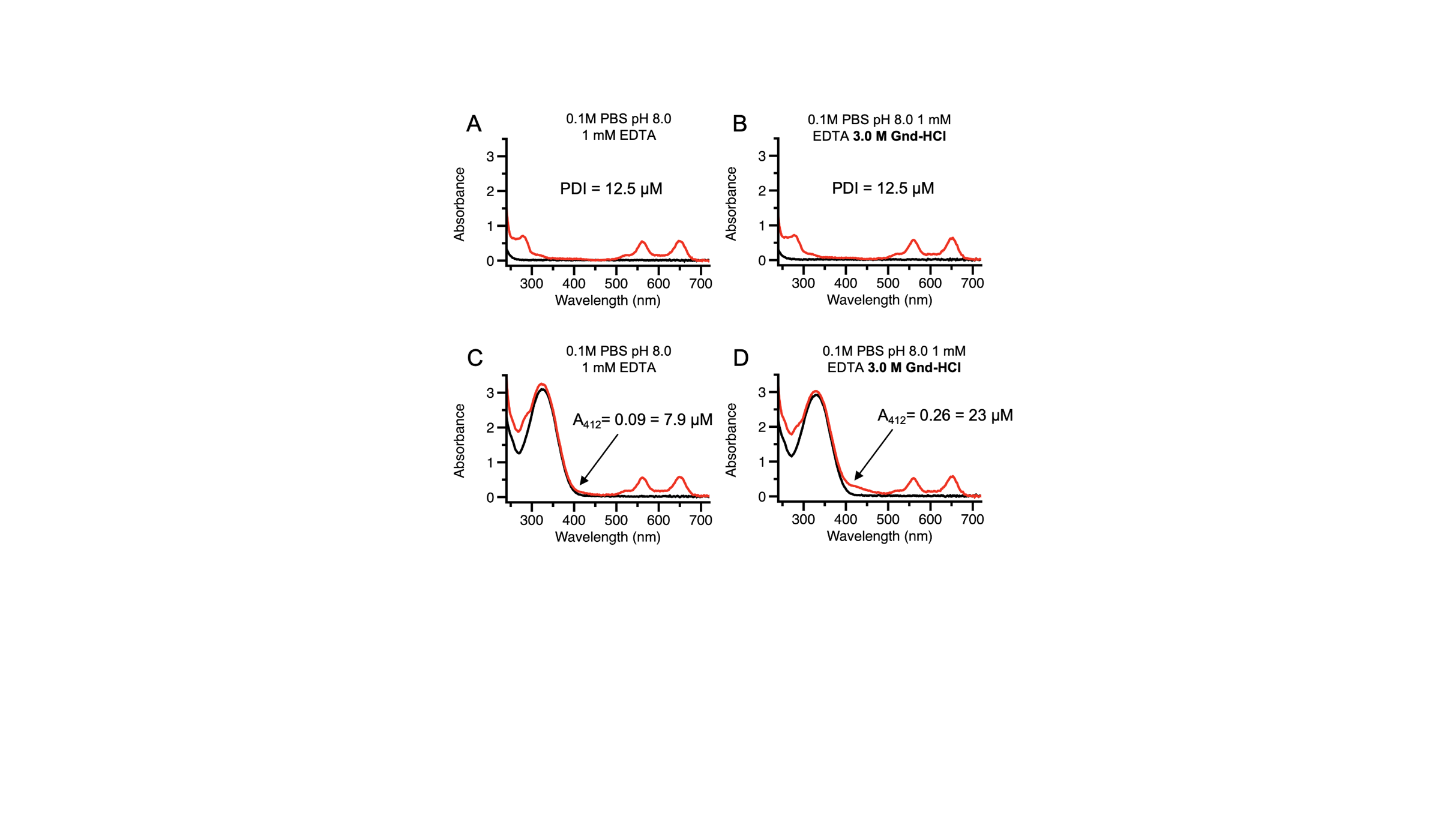
**

**Figure S5. Ellman’s reaction.** SEC-purified PDI 88/467 labeled with Atto-550 and 647N (12.5 μM) was solubilized in 0.1M PBS pH 8.0 1 mM EDTA in the absence (A) and presence (B) of 3M Gnd-HCl. Based on previous studies (2), this concentration of Gnd-HCl is sufficient to unfold PDI. The same concentration of protein was incubated for 20 minutes at room temperature in the dark with 0.1 mg/ml of Ellman's Reagent (DTNB, 5,5′-dithiobis(2-nitrobenzoic acid) solubilized in 0.1M PBS pH 8.0 1 mM EDTA in the absence (C) and presence (D) of 3M Gnd-HCl. To ensure all available cysteines reacted with DTNB, spectra for sample D were collected after 3 additional hours. Identical results were obtained. In this reaction, the thiolate form of the cysteine sulfhydryl group reacts with Ellman's reagent to form TNB^2−^, which absorbs light at 412 nm, A_412_ (indicated by the arrow). Spectra were recorded using a Nanodrop One (ThermoFisher) allowing quantification of TNB^2−^. The concentration of TNB^2−^ is calculated after subtraction of baseline using the Lambert-Beer law using a molar extinction coefficient of 11,400 M^−1^ cm^−1^ (4). These studies indicate that each PDI molecule (12.5 μM) contains two residues of cysteines (23 μM) that can react with DTNB. They also show that the reactivity of these cysteine residues is favored by denaturing PDI suggesting slow reactivity and/or inaccessibility in the folded state. PDI contains 6 cysteine residues, 4 in the active sites and 2 in the b’ domain. Previous structural studies have shown that cysteine residues in the b’ domain are buried and reduced (1,5). Our data are consistent with a model in which the 2 cysteines in the b’ are reduced whereas the cysteines of the active sites are oxidized.

**
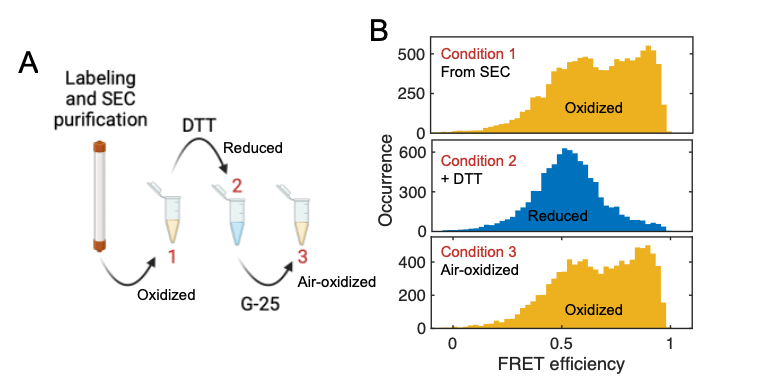
**

**Figure S6. Air-oxidation of PDI monitored by smFRET. A)** smFRET measurements were collected on the very sample of 88/467 that was progressively subjected to three different conditions. These are condition 1: right after SEC purification; condition 2: after addition of DTT (50 μM); condition 3: after removal of DTT by a G-25 desalting spin column followed by overnight incubation at room temperature in the dark. **B)** FRET histograms corresponding to conditions 1, 2, and 3. FRET histograms obtained for conditions 1 and 3 are identical, but different from condition 2. This proves that, after labeling and purification, the catalytic cysteines are oxidized and that FRET changes induced by DTT are reversible, as expected for a catalytically active enzyme.

**
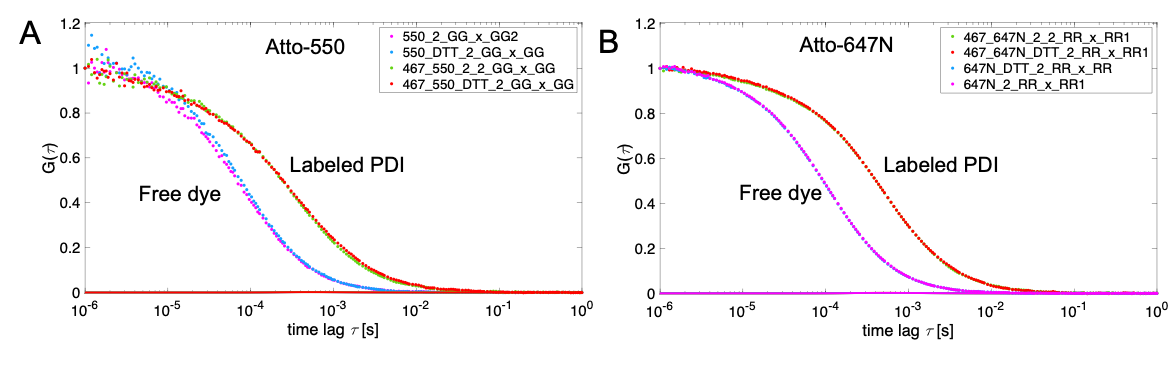
**

**Figure S7. Effect of DTT on the dye photo-physics.** Shown are the normalized autocorrelation functions for Atto-550 (A) and Atto-647N (B) channels. The molecular brightness of free and protein-bound is not affected by DTT (50 mM). Note how the diffusion time of the labeled PDI is slower compared to free dye, implying dyes are covalently linked to PDI and no free dyes are present in the PDI solutions.


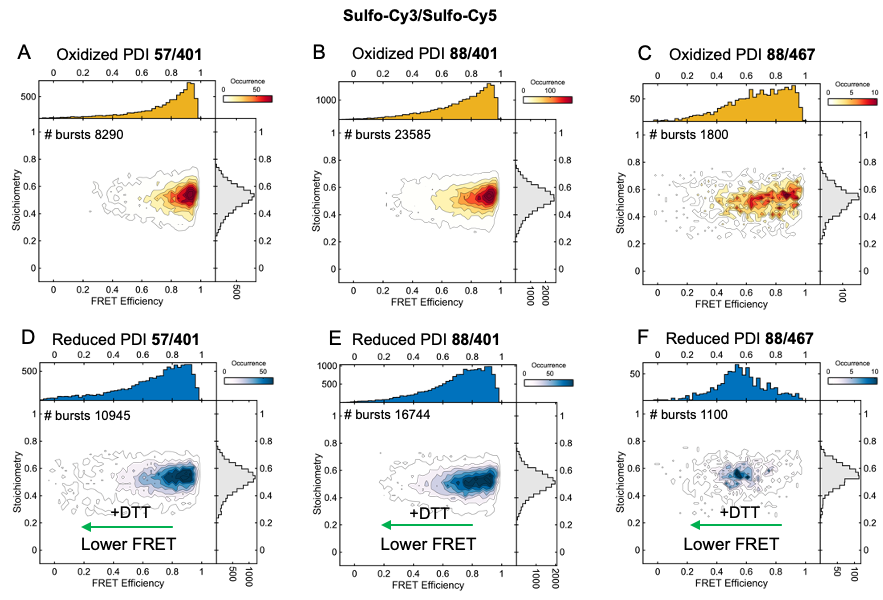
**Figure S8. FRET efficiency vs stoichiometry plots of PDI variants labeled with Cy dyes.** FRET efficiency vs stoichiometry plots of 57/401 **(A, D)**, 88/401 **(B, E)**, and 88/467 **(C, F)**, under non-reducing (top, yellow) and reducing conditions (bottom, blue) labeled with Cy dyes. Like in **Figure S3**, addition of DTT, while shifting E_ave_ towards lower FRET (green arrow line), does not affect stoichiometry, consistent with a monomeric enzyme undergoing redox-stimulated conformational changes.


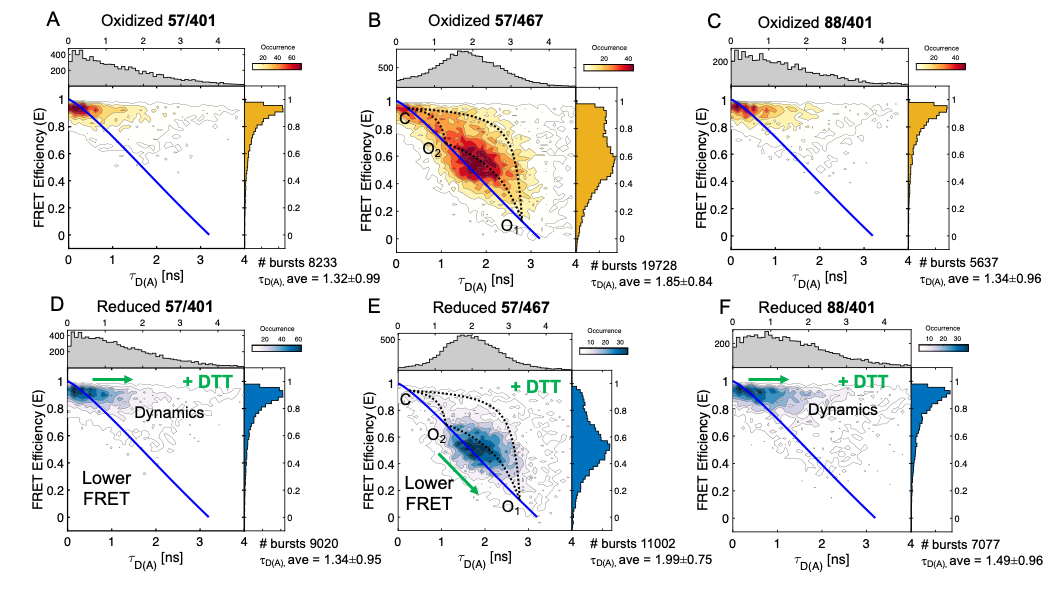


**Figure S9. FRET efficiency vs lifetime plots of PDI variants labeled with Atto dyes.** FRET efficiency vs lifetime plots of 57/401 **(A, D)**, 57/467 **(B, E)** and 88/401 **(C, F)** under non-reducing (top, yellow) and reducing (1 mM DTT) conditions (bottom, blue). Static FRET lines (solid blue lines) are shown in each plot. Due to high FRET, 57/401 **(A** and **D)** and 88/401 **(B** and **E)** show only hints of dynamics, which manifests as a small but significant deviation of the high FRET ensemble towards the right of the static FRET line. Also evident in these plots is the shift toward lower FRET induced by DTT. In contrast to 57/401 and PDI 88/401, but similar to PDI 88/467 (**Figure 2** of the main text), 57/467 **(C** and **F)** shows a very clear dynamic signature documenting dynamic exchange between closed (C, high FRET) and open (O, medium FRET) ensembles. These two ensembles are characterized by mean fluorescence lifetime values of ~0.25 and ~1.8 ns, respectively. The open ensemble of 57/467, similar to 88/467, is shifted toward the right of the static FRET line indicating fast dynamics between open states. Using the same methodology described in the main text, we identified O_1_ (1.1±0.2 ns) and O_2_ (2.8±0.3 ns), which are shown in the plot along the dynamic FRET lines (black dotted lines) that connect them. The green arrow line indicates a shift towards lower FRET in the presence of DTT.


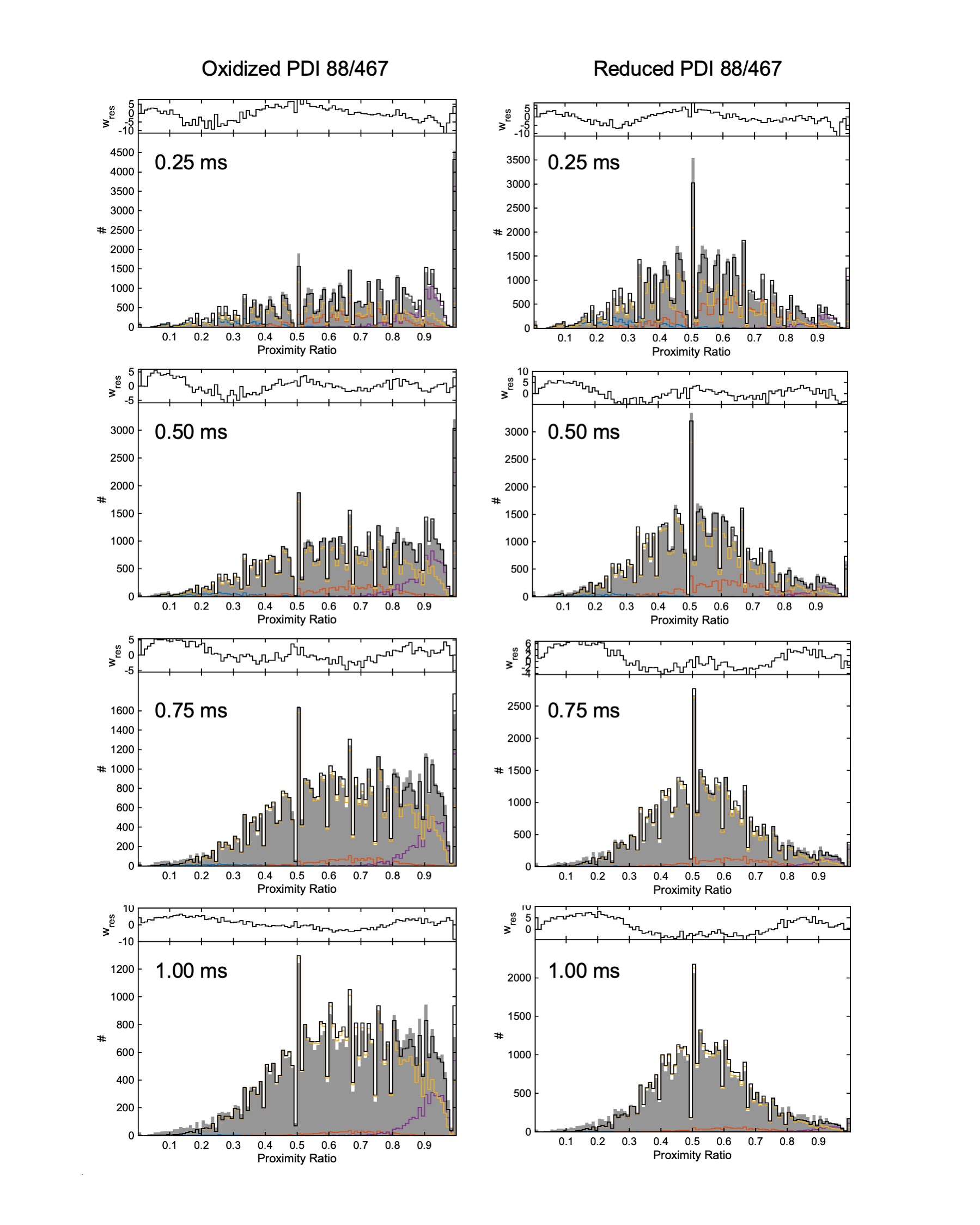
**Figure S10. PDA analysis of PDI 88/467.** PDA was performed on datasets binned at 0.25, 0.5, 0.75, and 1 ms. Photons from each burst were used to build a proximity ratio (PR) histogram. The resulting histogram was then fitted using a Monte Carlo approach for simulating the burst-wise histogram using a dynamic three-state model (3). To assess the robustness of the fit, PDA was repeated by systematically varying the initial value of the rate constants to 1, 0.5, and 0.75 ms^-1^ (min 0, max 10) while keeping the other settings identical. Corresponding weighted residuals are shown above each plot.

**
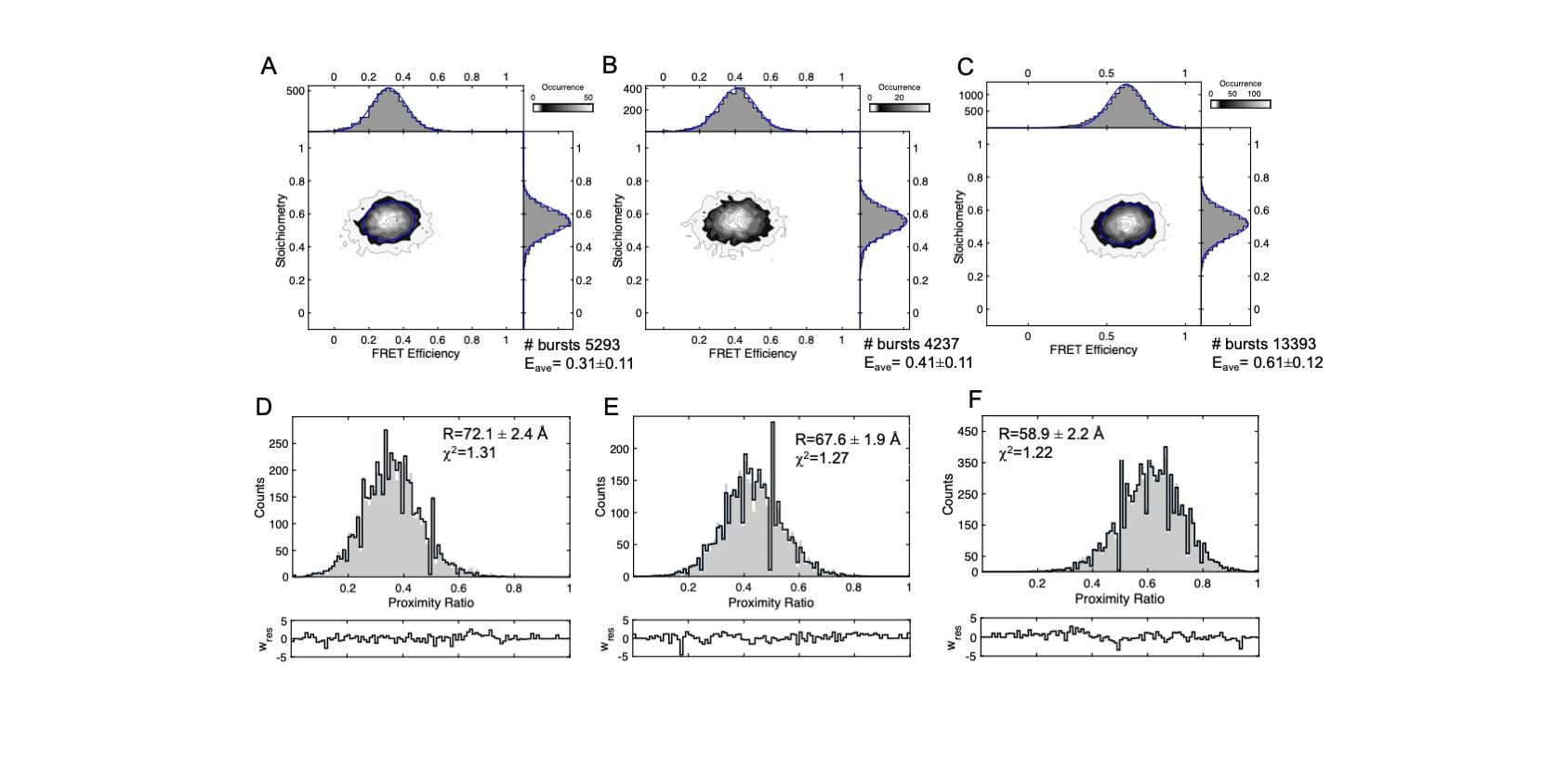
Figure S11. Analysis of static double-stranded DNA constructs.** Plots **(A, B** and **C)** and PDA **(D, E** and **F)** analysis of DNA duplexes with probes separated by 19 **(A)**, 17 **(B)** and 14 **(C)** base pairs. Single-stranded DNA molecules were purchased (IDT Inc., Coralville, LA) and fluorescent dyes (Atto550/647N) were attached to amino dT residues obtained by substituting T to iAmMC6T. dsDNA molecules were formed by hybridization. Experimental conditions are 100 pM in TBS-Tween 0.01%. FRET histograms best fit one Gaussian distribution (blue). Note how the standard deviation for static species is significantly smaller compared to values obtained in this work for PDI **(Table 1)**, supporting the view that PDI adopts multiple conformations in solution. PDA was performed on datasets binned at 1 ms. Photons from each burst were used to build a proximity ratio histogram. The resulting histogram was then fitted using a Monte Carlo approach for simulating the burst-wise histogram using one Gaussian. Corresponding weighted residuals are shown below each plot.


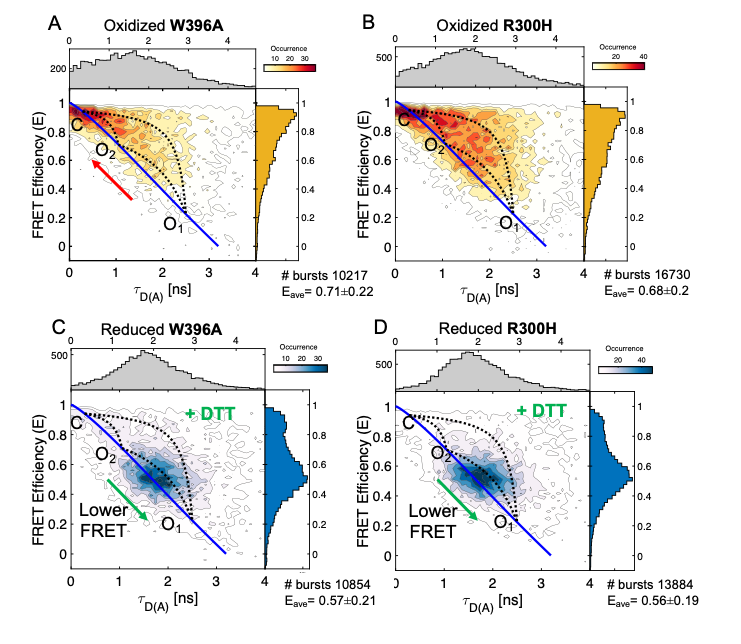


**Figure S12. FRET efficiency vs lifetime plots of PDI W396A and R300H variants labeled with Atto dyes.** FRET efficiency vs lifetime plots of active site variants under non-reducing (top, yellow) and reducing conditions (bottom, blue). Shown are static (solid blue lines) and dynamic (solid green lines) FRET lines connecting the FRET states. The lines were drawn as described in the main text. The position of C, O_1_ and O_2_ is indicated. The fraction of each population was obtained by PDA and is reported in **Table 1**. The red arrow line indicates a shift towards high FRET compared to PDI WT. The green arrow line indicates a shift towards lower FRET in the presence of DTT.


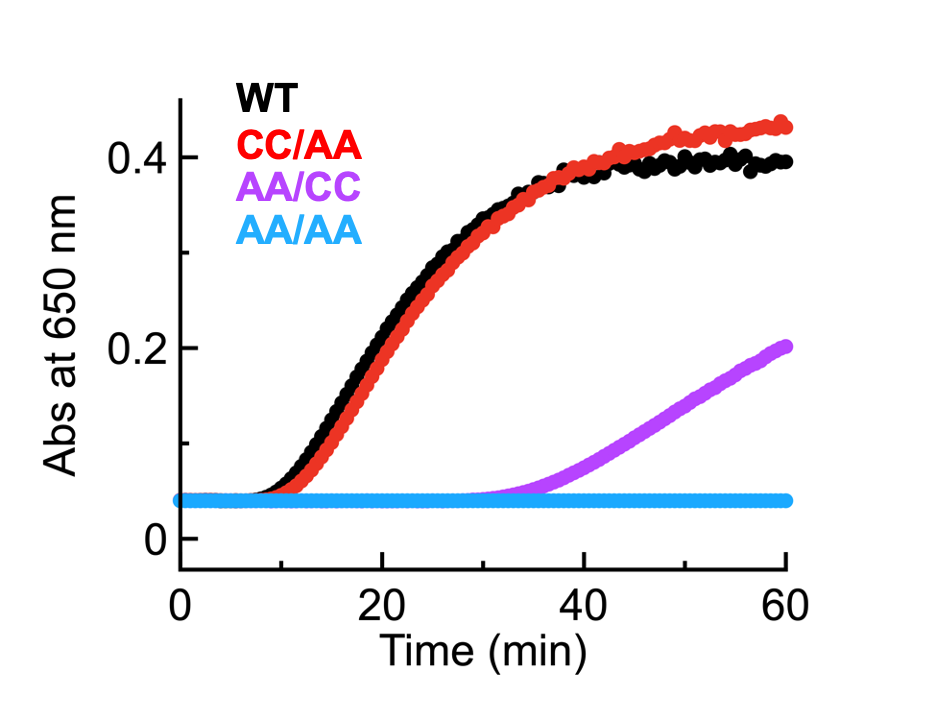


**Figure S13. Reductase activity of the 88/467 and active site mutants.** Reductase activity of PDI 88/467 (WT, black) and active site variants AA/AA (blue), CC/AA (red) and AA/CC (magenta) monitored by the insulin assay. Note how the catalytic activity of CC/AA is similar to PDI WT but different from AA/CC, whose catalytic activity is compromised. Among the two active sites, the one in the **a** domain is the most important for insulin reduction. AA/AA is catalytically inactive, as expected since it no longer contains cysteine residues in the active sites.


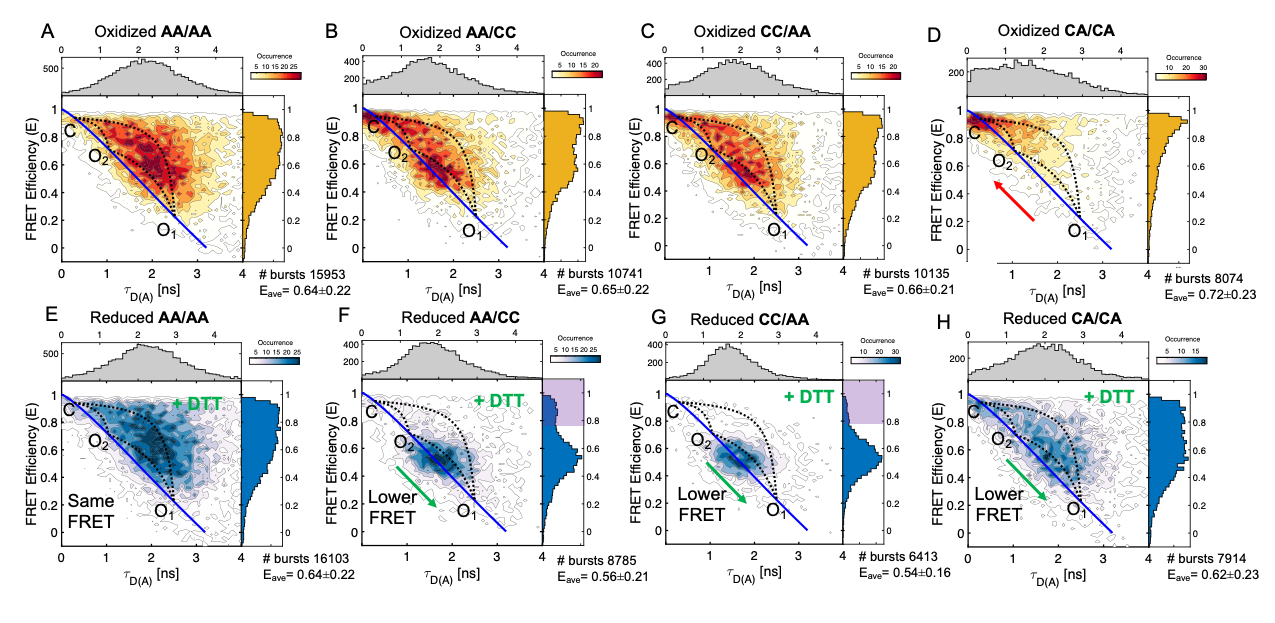
**Figure S14. FRET efficiency vs lifetime plots of PDI active site variants labeled with Atto dyes.** FRET efficiency vs lifetime plots of active site variants under non-reducing (top, yellow) and reducing conditions (bottom, blue). Shown are static (solid blue lines) and dynamic (solid green lines) FRET lines connecting the FRET states. The lines were drawn as described in the main text. The position of C, O_1_ and O_2_ is indicated. The fraction of each population was obtained by PDA and is reported in **Table 1**. The red arrow line indicates a shift towards high FRET compared to PDI WT. The green arrow line indicates a shift towards lower FRET in the presence of DTT.

**Table S1.** Anisotropy and quantum yield (Φ) values for singly labeled PDI mutants.

|  | **Atto 550** | | **Atto 647N** | |
| --- | --- | --- | --- | --- |
|  | **Anisotropy** | **Quantum yield** | **Anisotropy** | **Quantum yield** |
| **K57U** | 0.23±0.02 | 0.77±0.02 | 0.24±0.02 | 0.63±0.02 |
| **K88U** | 0.19±0.02 | 0.79±0.02 | 0.18±0.02 | 0.65±0.02 |
| **K401U** | 0.22±0.02 | 0.78±0.02 | 0.22±0.02 | 0.64±0.02 |
| **K467U** | 0.21±0.02 | 0.79±0.02 | 0.21±0.02 | 0.65±0.02 |

Experimental conditions are 100 mM potassium phosphate (pH 7.4), 2 mM EDTA, at 25°C. For anisotropy determination, the concentration of PDI was 10 nM, ex 540/em580 for Atto-550 and ex640/em680 for Atto-647N, ex/em slits 1 and 14 nm, respectively. The results represent the average of two independent determinations. For quantum yield determination Rhodamine 110(Φ=0.89±0.02) in ethanol was used as a standard, for free dyes in buffer, Atto-550(Φ=0.79±0.02) and Atto-647N (Φ=0.64±0.02) were also experimentally determined.

**Table S2**. Subpopulation specific fluorescence lifetime analysis of oxidized and reduced 88/467

|  |  | | | | |
| --- | --- | --- | --- | --- | --- |
|  | **τ1 (ns)** | **f1** | **τ2 (ns)** | **f2** | **Χ^2^** |
| **oxidized 88-467** |  |  |  |  |  |
| **1 exp** | 2.23 ± 0.08 | 1 | - | - | 9.69 |
| **2 exp** | 2.91 ± 0.15 | 0.44 | 0.90 ± 0.06 | 0.56 | 1.32 |
|  |  |  |  |  |  |
| **reduced 88-467** |  |  |  |  |  |
| **1 exp** | 2.04 ± 0.08 | 1 | - | - | 14.58 |
| **2 exp** | 2.72 ± 0.15 | 0.42 | 0.91 ± 0.06 | 0.58 | 1.32 |

**References**

1. Wang, C., Yu, J., Huo, L., Wang, L., Feng, W., and Wang, C. C. (2012) Human protein-disulfide isomerase is a redox-regulated chaperone activated by oxidation of domain a'. *J Biol Chem* **287**, 1139-1149

2. Chinnaraj, M., Barrios, D. A., Frieden, C., Heyduk, T., Flaumenhaft, R., and Pozzi, N. (2021) Bioorthogonal Chemistry Enables Single-Molecule FRET Measurements of Catalytically Active Protein Disulfide Isomerase. *Chembiochem* **22**, 134-138

3. Schrimpf, W., Barth, A., Hendrix, J., and Lamb, D. C. (2018) PAM: A Framework for Integrated Analysis of Imaging, Single-Molecule, and Ensemble Fluorescence Data. *Biophys J* **114**, 1518-1528

4. Riddles, P. W., Blakeley, R. L., and Zerner, B. (1983) Reassessment of Ellman's reagent. *Methods Enzymol* **91**, 49-60

5. Wang, C., Li, W., Ren, J., Fang, J., Ke, H., Gong, W., Feng, W., and Wang, C. C. (2013) Structural insights into the redox-regulated dynamic conformations of human protein disulfide isomerase. *Antioxid Redox Signal* **19**, 36-45
